# Chemical Synthesis of Δ-4,5 Unsaturated Heparan Sulfate Oligosaccharides for Biomarker Discovery

**DOI:** 10.1021/acs.orglett.4c00596

**Published:** 2024-03-18

**Authors:** Apoorva Joshi, Pradeep Chopra, Andre Venot, Geert-Jan Boons

**Affiliations:** † Complex Carbohydrate Research Center, University of Georgia, 315 Riverbend Road, Athens, Georgia 30602, United States; ‡ Department of Chemistry, University of Georgia, Athens, Georgia 30602, United States; § Department of Chemical Biology and Drug Discovery, Utrecht Institute for Pharmaceutical Sciences, and Bijvoet Center for Biomolecular Research, Utrecht University, Universiteitsweg 99, 3584 CG Utrecht, The Netherlands

## Abstract

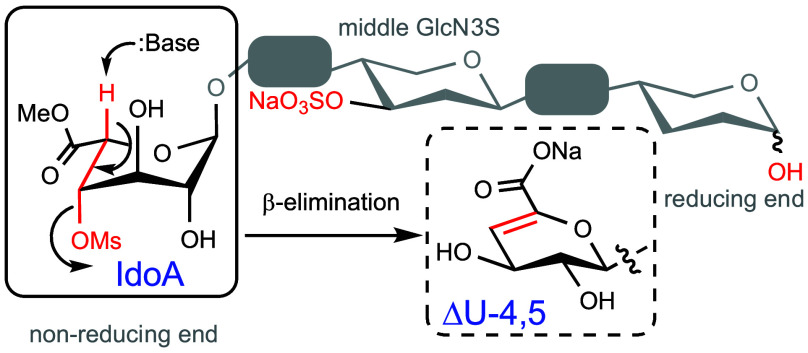

A methodology is described that can provide heparan sulfate oligosaccharides having a Δ4,5-double bond, which are needed as analytical standards and biomarkers for mucopolysaccharidoses. It is based on chemical oligosaccharide synthesis followed by modification of the C-4 hydroxyl of the terminal uronic acid moiety as methanesulfonate. This leaving group is stable under conditions used to remove temporary protecting groups, *O*-sulfation, and hydrogenolysis. Treatment with NaOH results in elimination of the methanesulfonate and formation of a Δ4,5-double bond.

Heparan sulfates (HS) are sulfated biopolymers ubiquitously presented on the surface, in the extracellular matrix, and in the basement membrane of almost all animal cells.^1^ The biosynthesis of HS involves the formation of linear polymers of alternating 1,4-linked d-glucuronic acid (GlcA) and *N*-acetyl-d-glucosamine (GlcNAc) that are modified by a series of enzymatic transformations including *N*-deacetylation and *N*-sulfation, epimerization of GlcA to l-iduronic acid (IdoA), and sulfation of the C-2 hydroxyl of the uronic acids and the C-6 and C-3 hydroxyl of glucosamine moieties.^1,2^ Epimerization and sulfation proceed only partially, resulting in considerable structural variability and the creation of specific epitopes that can interact with a multitude of regulatory proteins such as chemokines and cytokines, growth factors, morphogens, coagulation factors, and cell adhesion proteins.^3^ Interactions of HS with HS-binding proteins are important for many biological processes, such as the formation of signaling complexes, controlling stem cell renewal, as well as determining cell fate, proliferation, or differentiation to specific lineages.^1−3^ Alterations in expression and degradation of HS has been implicated in neural dysfunction, Alzheimer’s disease, atherosclerosis, rheumatoid arthritis, renal fibrosis, and lysosomal storage disorders.^4^

Heparin, which is a member of the HS family that has a higher level of sulfation, is widely used as an anticoagulant agent to avoid blood clotting.^5^ Modified heparins that lack anticoagulation activity have enormous therapeutic potential for diseases, such as cancer, diabetes, infectious diseases such as SARS-CoV-2, wound-healing, and inflammatory and neurological conditions.^6−9^ Despite its clinical use since 1936, the structural complexity of heparin and modified heparins is still poorly understood.^10^

To understand the biology of HS and realize the extraordinary potential of next-generation heparins, methods are needed to determine structures of HS and heparin.^11^ A commonly applied analytical approach is based on hydrolysis of heparin or HS into smaller fragments by heparin lyases from *Flavobacterium heparinum* or *Bacteroides eggerthii* followed by identification of the resulting saccharides by liquid chromatography mass spectrometry (LC-MS),^12^ ion mobility mass spectrometry (IM-MS),^13^ or capillary zone electrophoresis mass spectrometry (CZE-MS).^14^ Heparin lyases cleave specific glycosidic bonds between glucosamine and uronic acid, thereby generating β-elimination products having a Δ4,5-unsaturated uronic acid moiety (Figure 1).^15,16^ A combination of lyases I/II/III almost fully digests HS and heparin to provide disaccharides, and by matching chromatographic properties to well-defined standards, compositional information is commonly obtained.^11,12^ It has, however, been difficult to identify 3-*O*-sulfated disaccharides due to the lack of appropriate analytical standards. We addressed this deficiency by chemically synthesizing a series of 3-*O*-sulfated tetrasaccharides having different patterns of *N*-, 2-*O*-, and 6-*O*-sulfation that were treated with a mixture of heparin lyase I, II, and III to prove a comprehensive series of lyase products having a 3-*O*-sulfate. The compounds were derivatized with fluorescent 2-aminoacridone (AMAC) via reductive amination to give, after purification by reverse phase HPLC, eight 3-*O*-sulfated disaccharide standards. These compounds made it possible, for the first time, to comprehensively determine compositions of clinical-grade, unfractionated, and low-molecular-weight heparins.^10^

**Figure 1:**
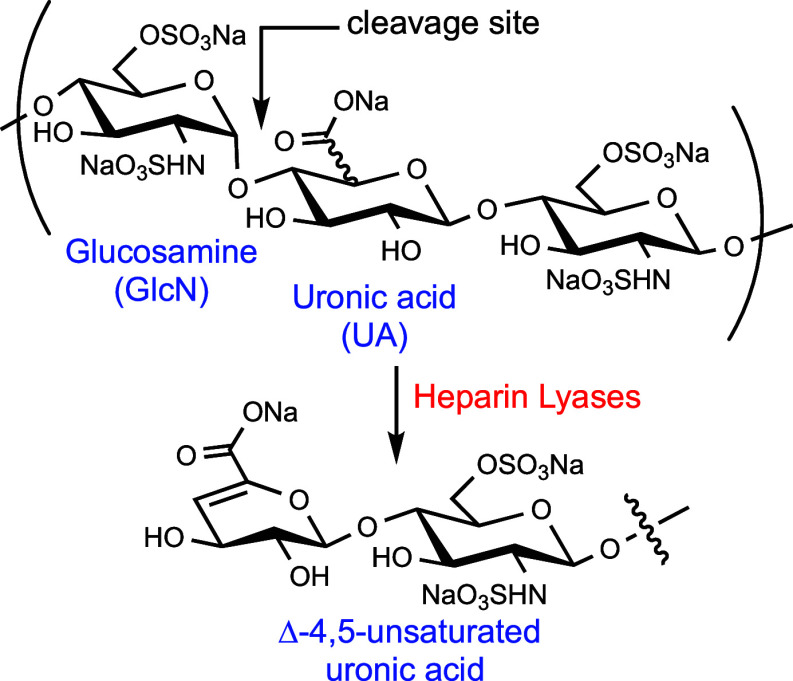
Heparan sulfate digestion by heparin lyases enzymes.

The use of discrete lyases results in the formation of larger structures that, in principle, can provide sequence information. Well-defined lyase products larger than disaccharides are required to develop methods to identify such compounds. Treatment of heparin with lyases I/II/III leaves some larger products, and it seems 3-*O*-sulfation can inhibit full digestion.^17,18^ Standards are also needed to identify these compounds. Well-defined lyase products are also needed to develop biomarkers for mucopolysaccharidoses (MPS).^19^ These inherited diseases result in lysosomal accumulation of partially degraded GAGs, such as HS, due to mutations in metabolic enzymes.^20^ Identification of the structures of these partially degraded GAGs is expected to provide biomarkers for early disease diagnosis.^19−21^

Despite advancements in generating well-defined disaccharide lyase products, there are no general methods to generate longer structures having a terminal Δ4,5-unsaturated uronic acid.^22^ A small number of tetrasaccharides having a Δ4,5-unsaturated uronic acid moiety has been prepared by controlled degradation of unfractionated (UFH) and low-molecular-weight (LMWH) heparin^23^ and HS octasaccharides^24^ by a mixture of lyases followed by multistep chromatographic purification. This approach is, however, not suitable for generating oligosaccharides larger than tetrasaccharides that have multiple cleavage sites. The chemical synthesis of this class of compounds is complicated by the incompatibility of the Δ4,5-double bond with the removal of benzyl ethers that are commonly employed as a permanent protecting group for the chemical synthesis of HS-oligosaccharides.

Here, we report a chemical approach that can provide panels of HS hexasaccharides bearing a Δ4,5-double bond and include compounds having a 3-*O*-sulfate (Figure 2). To develop the synthetic technology, we selected hexasaccharide **1** and its positional isomer **2** as the synthetic targets. Technologies to identify HS metabolites is still in its infancy, and precise structures of lyase cleavage products and biomarker for MPS diagnosis remain to be discovered. Compounds such as **1** and **2** are, however, expected to be partial HS degradation products that may occur in specific MPS diseases. In this respect, 3-*O*-sulfation is known to confer resistance to lyase-mediated degradation, and compounds **1** and **2** contain structural elements typical of 3-*O*-sulfation.^17,18^ Furthermore, lysosomal degradation of HS occurs by exo-acting enzymes starting from the nonreducing end and must act sequentially to fully break down HS oligosaccharide chains.^19^ Thus, a reduction of activity of a glycosidase or sulfatase, as in MPS diseases, is expected to result in the formation of larger oligosaccharide structures. The synthetic methodology employs modular disaccharide building blocks, such as **3**–**6**, that have selectively removable levulinoyl (Lev) esters at positions that ultimately need sulfation. Furthermore, it includes disaccharide donors such as **4** and **5** that have a 2-naphthylmethyl (Nap) ether at the C-3 hydroxyl of GlcN that, after oligosaccharide assembly, can selectively be removed to give a hydroxyl for sulfation. The C-4′ hydroxyl of the building blocks is protected as 9-fluorenylmethyl carbonate (Fmoc), which can selectively be removed by a hindered base to give a glycosyl acceptor for further glycosylations. After oligosaccharide assembly, removal of the Fmoc gives an alcohol that can be modified by the leaving group, methanesulfonate (Ms).^25,26^ It was found that the latter moiety is sufficiently stable under conditions used to remove the Lev ester and Nap ether, installation of *O*-sulfates, and global deprotection by hydrogenation over palladium/carbon (Pd/C). Treatment of the resulting compounds with base was expected to result in elimination of the methanesulfonate to install the Δ4,5-double bond. IdoA was chosen as the terminal uronic acid moiety since it places the leaving group at C-4 and proton at C-5 in *anti*-configuration, which is favorable for β-elimination.^27,28^ As a final step, *N*-sulfates are selectively introduced by using a sulfur trioxide–pyridine (SO_3_·Py) complex under basic conditions.

**Figure 2:**
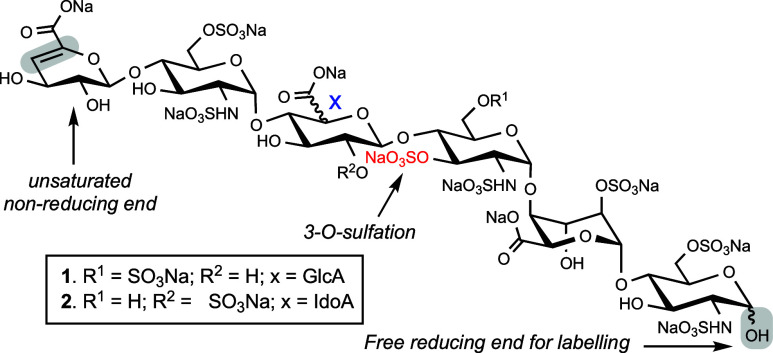
Target hexasaccharides **1** and **2**.

The modular disaccharide building blocks **3**–**6** (Scheme 1) were prepared on a large scale from properly protected 2-azido-2-deoxy-glucopyranoside, thioethyl glycosyl, and thioethyl idosyl donors following reported procedures (see Supporting Information).^29^ The preparation of hexasaccharides **1** and **2** is described in Scheme 1 and 2. Thus, a triflic acid (TfOH)-mediated glycosylation of donors **4** and **5** with glycosyl acceptor **3** gave tetrasaccharides **7** and **8**, respectively, as only the α-anomer, which was confirmed by the small *J*_1,2_ coupling constant (∼4.0 Hz) and ^13^C chemical shifts of C-1 (∼97.0 ppm) in nuclear magnetic resonance (NMR) spectra. The Fmoc protecting group of **7** and **8** was removed using triethylamine (Et_3_N) in dichloromethane (DCM), and the resulting glycosyl acceptors were coupled with glycosyl donor **6** to obtain fully protected hexasaccharides **9** and **10**, respectively (Scheme 2). NMR analysis confirmed the α-anomeric configuration of the products. Compounds **9** and **10** were treated under standard conditions to remove the Fmoc protecting group, and the resulting C-4 hydroxyl was reacted with methanesulfonyl chloride (MsCl) in pyridine to provide **11** and **12** in yields of 65% and 59%, respectively.

**Scheme 1:**
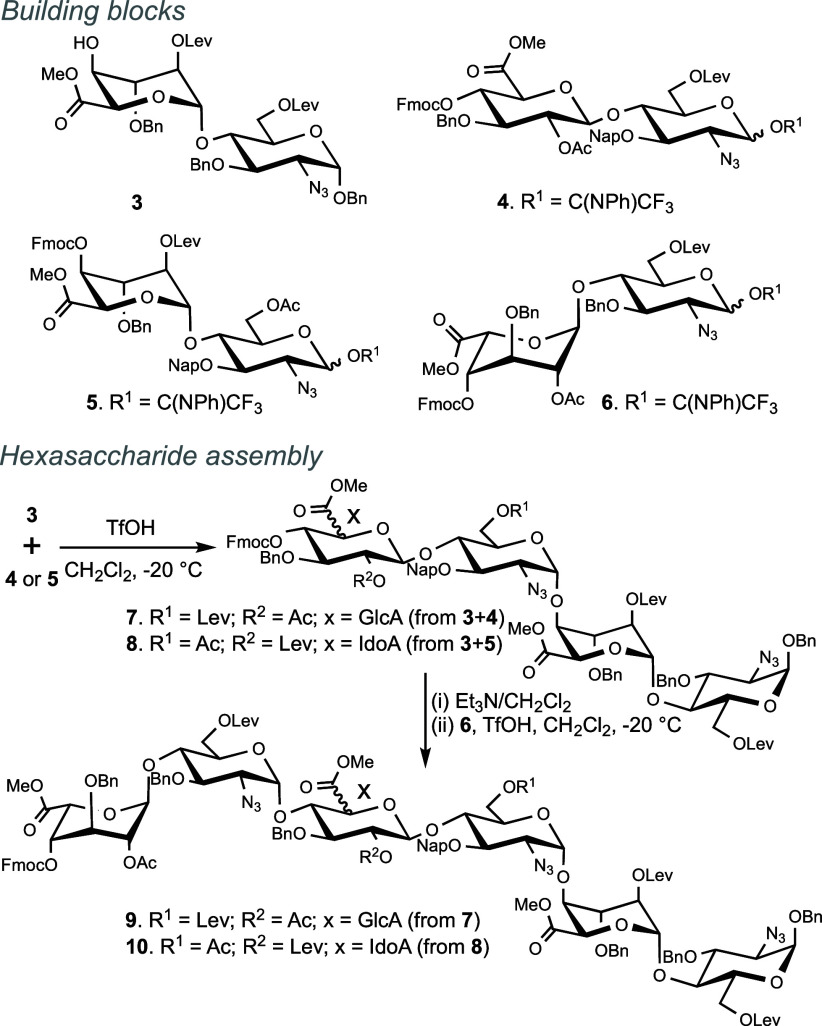
Disaccharide Building Blocks for Modular Synthesis and Assembly of Protected Hexasaccharides

**Scheme 2:**
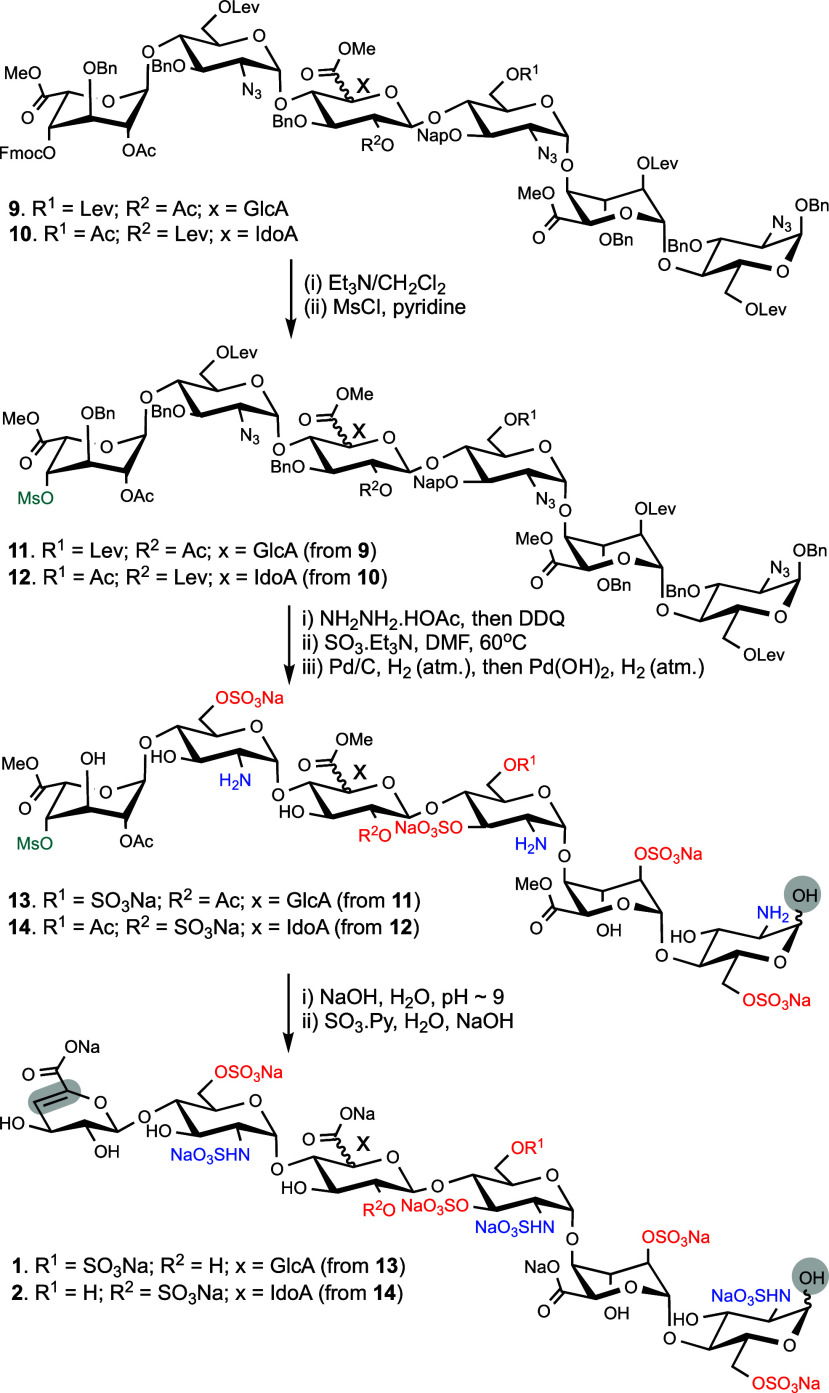
Preparation of Hexasaccharides **1** and **2**

Next, the Lev esters of **11** and **12** were removed by treatment with hydrazine acetate in a mixture of toluene/ethanol followed by oxidative removal of the Nap ether using 2,3-dichloro-5,6-dicyano-1,4-benzoquinone (DDQ) in a mixture of DCM/phosphate buffer saline. The resulting hydroxyls were sulfated using the sulfur trioxide–triethylamine (SO_3_·Et_3_N) complex at elevated temperature (60 °C) for 16 h. After confirming of the formation of the fully sulfated products by high-resolution electrospray ionization–mass spectrometry (ESI-MS), the reaction mixtures were purified by size-exclusion chromatography (SEC) over Sephadex LH-20 and then subjected to sodium exchange over Dowex [Na^+^] resin. A two-step catalytic hydrogenation, which involved first hydrogenation over Pd/C in a mixture of *tert*-butanol/water to reduce azido moieties into amine, followed by removal of benzyl ethers by hydrogenation over palladium hydroxide/carbon (Pd(OH)_2_/C), gave compounds **13** and **14**. Next, attempts were made to perform a β-elimination of the mesylate. First, the elimination was carried out using the hindered base 1,8-diazabicyclo[5.4.0]undec-7-ene (DBU). Although it resulted in a clean elimination product formation, the resulting SO_3_·DBU salts were very difficult to exchange to sodium using Dowex [Na^+^] resin.

The use of aqueous sodium hydroxide (0.1 M, NaOH) for an extended period resulted in elimination, deacetylation, and methyl ester hydrolysis. Finally, *N*-sulfation was performed using SO_3_·Py in H_2_O in the presence of NaOH, which, upon purification by SEC over Biogel P-2 column followed by sodium exchange using Dowex [Na^+^] resin, gave the targeted hexasaccharides **1** and **2** in yields of 62% and 65%, respectively.

The target hexasaccharides **1** and **2** were obtained in quantities ranging from 6 to 7 mg and were fully characterized by ESI-MS and NMR. ^1^H and ^13^C resonances were assigned by 1D and 2D NMR experiments. The sites of Δ4,5-unsaturation were confirmed by downfield shift of H-4 ∼5.6 ppm and C-4 ∼107.7 ppm, and sites of sulfation were confirmed by a downfield shift of ring carbons (∼4 ppm) and by downfield shifts of ring protons (∼0.5 ppm). Specifically, 3-*O*-sulfation resulted in a downfield shift of GlcN_H-2_ and showed a typical pattern in ^1^H–^13^C heteronuclear single quantum coherence spectroscopy (HSQC) NMR experiment.^18^

In conclusion, a synthetic methodology is described that can provide analytical HS standards having Δ4,5-unsaturation. It is based on chemical oligosaccharide assembly using modular disaccharide building blocks followed by modification of the C-4 hydroxyl of the terminal uronic acid as a methanesulfonate. It was found that this leaving group is sufficiently stable to chemical conditions to remove Lev esters and Nap ethers to give alcohols that can be selectively sulfated and subjected to hydrogenation to remove permanent benzyl ethers and reduce azides to amines. Treatment of the resulting compounds with aqueous NaOH resulted in elimination of the methanesulfonate and installation of an Δ4,5-double bond with concomitant cleavage of esters. Finally, selective *N*-sulfation under basic conditions gave the target compounds **1** and **2**.

Chemical synthesis offers the advantages of scalability and analytical purity and can, in principle, provide any possible sulfation pattern.^30−33^ The synthetic approach described here can provide panels of HS oligosaccharides having a Δ4,5-unsaturated uronic acid moiety and different patterns of sulfation by selecting modular disaccharides having different patterns of Lev esters. It includes compounds with 2-*O*-sulfated Δ4,5-unsaturated uronic acid (UA) by using a building block that has a Lev ester at C-2 and a mesylate at C-4 of IdoA. It can also provide 3-*O*-sulfated derivatives that are rare but are important for many biological properties of HS and heparin.^34^ 3-*O*-sulfation provides some resistance to lyase degradation, and its presence is expected to provide larger lyase products such as in compounds **1** and **2**.^17,18^ Well-defined lyase products are expected to facilitate the development of analytical methods for sequencing of heparin and HS and may lead to the identification of biomarkers for various MPS.^19^ In this respect, heparin/HS possess unparalleled levels of structural complexity and often occur in many isomeric forms that cannot be resolved by current analytical methods.^1^ Analytical standards, in particular lyase products, are needed to resolve these technical hurdles.^22^ Here, we prepared two isomeric compounds that will facilitate the identification of such compounds. The free reducing end of the oligosaccharides make it possible to introduce a ^13^C tag, such as aniline or AMAC,^10,19^ to give compounds that can be used as internal standards to facilitate quantification, which is important for use as biomarkers. It is to be expected that the chemical approach described here can provide a wide range of HS lyase products and can be applied to obtain other GAG standards, such as those derived from chondroitin sulfate that have also been implicated in MPS subtypes.

## Data Availability

The data underlying this study are available in the published article and its online Supporting Information

## Supporting Information Available

Complete experimental and spectral characterization (NMR and MS) details and copies of ^1^H and ^1^H -^13^C HSQC NMR spectra of all new compounds (PDF)
