# Supplementary material for: Chemical Synthesis of Δ-4,5 Unsaturated Heparan Sulfate Oligosaccharides for Biomarker Discovery
Source: Org Lett. 2024 Mar 18;26(12):2462–6. doi: 10.1021/acs.orglett.4c00596 (PMC10985652; doi:10.1021/acs.orglett.4c00596)

Supporting Information

Chemical Synthesis of Δ-4,5 Unsaturated Heparan Sulfate Oligosaccharides for Biomarker Discovery

Apoorva Joshi, Pradeep Chopra, Andre Venot, and Geert-Jan Boons*

Table of Contents

1. General Procedures………………………………………………………………… S3
2. Experimental Section………………………………………………………………. S9
3. References…………………………………………………………………………….. S24
4. Spectral Data………………………………………………………………………….. S25

General Procedures[1-4]

All reagents and solvents were purchased from commercial sources and were used without further purification. Acid washed molecular sieves (4Å) were flame activated in vacuo. All moisture sensitive reactions were carried out under an argon atmosphere. Reactions were monitored using TLC on aluminium-backed plates coated with Silica Gel 60 F_{254} (E. Merck) and visualized by UV light (254 nM) where applicable, and by application of 5% H_2SO_4 in EtOH or with a solution of $(NH_4)_6Mo_7O_{24}.H_2O$ (25.0 gL^{-1}) in 10% H_2SO_4 in EtOH, as appropriate, and heating. Column chromatography was performed on silica gel G60 (Silicycle, 60-200 µm, 60 Å), or on Bondapak C-18 (Waters). Size exclusion chromatography was carried out on a Sephadex™ LH-20 using MeOH/DCM (1/1, v/v) as elution system. Desalting was carried out on a Bio-Gel P-2 gel column using H_2O as elution. ^1H and ^{13}C (data obtained from F1 dimension in the ^1H-^{13}C HSQC experiment) NMR spectra were recorded on Varian Mercury 300 MHz, Varian INOVA 500 MHz, 600 MHz, Georgia Research Alliance (GRA) University of Georgia 800 MHz spectrometers. Chemical shifts are reported in parts per million (ppm) relative to tetramethylsilane (TMS) as the internal standard. NMR data is presented as follows: chemical shift, multiplicity (s = singlet, d = doublet, t = triplet, dd = doublet of doublet, m = multiplet and/or multiple resonances), integration, coupling constant in Hertz (Hz). Structural assignments were made with additional information from gCOSY and gHSQC experiments. Mass spectra were recorded on an ABISciex 5800 MALDi-TOF-TOF or Shimadzu LCMS-IT-TOF mass spectrometer. The matrix was used was 2, 5-dihydroxy-benzoic acid (DHB) and Ultamark 1621 as the standard.

General Glycosylation Procedure for Synthesis of Disaccharides. Glycosyl thioethyl donor (1.2 equiv. based on acceptor) and 2-azido-2-deoxy-D-glucopyranoside acceptor (1.0 equiv.) were combined in a flask, co-evaporated with toluene (3 × 10 mL) and dissolved in DCM to maintain a

concentration of 0.02 M (based on donor). Powdered freshly activated 4 Å molecular sieves (weight of sieves equal to the combined weight of donor and acceptor) were added, and the mixture was stirred for 30 min at ambient temperature and then cooled to -30 °C (acetone/dry ice mixture). NIS (1.5 equiv.) and TfOH (0.15 equiv.) were added to the mixture and stirring was continued until TLC indicated disappearance of glycosyl donor (~20 min). The reaction mixture was allowed to warm to +5 °C and then quenched by the addition of 5µl pyridine. The mixture was filtered, the filtrate was decolorized with aqueous $Na_2S_2O_3$ (10%, 10 mL). The mixture was extracted with DCM, and the combined aqueous layers were back-extracted with DCM. The combined organic layers were dried ($MgSO_4$) and filtered. The filtrate was concentrated in vacuo, and the residue was purified by silica gel column chromatography using a stepwise gradient of toluene and EtOAc to give a pure disaccharide.

General Procedure for Benzylidene Acetal Cleavage of Disaccharides. A solution of a disaccharide in a mixture of DCM:TFA:H_2O (0.06 M, 10/1/0.1, v/v/v) was stirred at ambient temperature for 30 min. The reaction mixture was quenched with saturated aqueous $NaHCO_3$. The mixture was extracted with DCM. The combined organic layers were dried ($MgSO_4$) and filtered. The filtrate was concentrated in vacuo, and the residue was purified by silica gel column chromatography using a mixture of toluene and EtOAc to give pure diol.

General Procedure for TEMPO/BAIB-Mediated Oxidation and Esterification by Diazomethane. To a vigorously stirred solution of the diol (0.3 M) in a mixture of DCM:H_2O (2/1, v/v) was added TEMPO (0.2 equiv.) and BAIB (2.5 equiv.). Stirring was continued until TLC indicated complete conversion of the starting material to a spot of lower R_f. The reaction mixture was quenched by the addition of aqueous $Na_2S_2O_3$ (10%, 10 mL). The mixture was extracted with EtOAc, and the combined aqueous layers were back-extracted with EtOAc. The combined organic

layers were dried ($MgSO_4$) and filtered, and the filtrate was concentrated in vacuo. The oily residue was dissolved in THF (0.1 M) and treated with an excess of freshly prepared ethereal solution of diazomethane until the reaction mixture stayed yellow. The excess diazomethane was quenched by the addition of AcOH until the reaction mixture became colorless. The mixture was concentrated in vacuo and the residue was purified by silica gel column chromatography to yield a methyl ester.

General Procedure for Mesyl (Ms) Group Introduction. To a solution of starting material (0.03 M) in pyridine at 0 °C was added methanesulfonyl chloride (Mesyl, MsCl, 5 equiv.). The reaction mixture was brought to room temperature and stirring was continued until TLC indicated complete consumption of the starting material (~2 h). After quenching the reaction with MeOH (50 μL), the mixture was diluted with DCM (50 mL) and washed with saturated aqueous $NaHCO_3$ (2 × 50 mL) and brine (50 mL). The organic phase was dried ($MgSO_4$) and filtered, and the filtrate was concentrated in vacuo. The residue was purified by over silica gel chromatography using a gradient of hexanes and EtOAc to give pure product.

General Procedure for Cleavage of Lev Esters. To a solution of hexasaccharide in a mixture of ethanol and toluene (2/1, v/v, 5 mL for 150 mg) was added anhydrous hydrazine acetate (5 equiv. per Lev group). The reaction mixture was stirred until TLC indicated disappearance of starting material (~2 h). The reaction mixture was diluted with EtOAc (10 mL), washed with and brine (2 × 5 mL), dried (MgSO4), and filtered. The filtrate was concentrated, and the residue was purified by silica gel column chromatography using a gradient of hexanes or toluene and EtOAc to afford pure product.

General Procedure for Nap Removal. To a solution of starting material in a mixture of DCM and phosphate buffer (0.1M, pH~7.4) (20/1, v/v) was added DDQ (1 equiv.) at 0 °C. Two

additional portions of DDQ (1 equiv. each) were added after 1 h and 2 h, respectively at 0 °C (Ice-bath ~ 0 °C). The reaction mixture was stirred at room temperature until TLC indicated completion (~2 h). The reaction was quenched by the addition of saturated aqueous $NaHCO_3$. The mixture was extracted with DCM (2 × 10 mL). The combined organic layers were dried ($MgSO_4$) and filtered. The filtrate was concentrated in vacuo, and the residue was purified by preparative TLC using a mixture of hexanes and EtOAc as solvent system to give pure product.

General Procedure for *O*-Sulfation. To a solution of the starting material in DMF (1.0 mL for 0.02 mmol) was added $SO_3.Et_3N$ complex (20.0 equiv. per OH) was added and stirred at 60 ºC in an oil bath. After 48h, TLC (EtOAc/MeOH/H_2O 7/2/1, v/v/v) indicates reaction completion, the reaction mixture was cooled down to rt and MeOH (1.0 mL) was added to quench excess complex. The mixture was concentrated in vacuo (water bath temperature ~ 20 °C), and the residue was used for the next reaction.

General Procedure for Global Debenzylation. Palladium on carbon (Pd/C, 10%, 1.5 times the weight of starting material) was added to a solution of the starting material in *t*-BuOH and H_2O (1/1, v/v, 1 mL for 5 mg). The mixture was placed under an atmosphere of hydrogen, and the progress of the reaction was monitored by TLC ($CHCl_3$/CH_3OH/H_2O 60/40/10, v/v/v; EtOAc/pyridine/water/AcOH, 3/5/3/1, v/v/v). The hydrogenation was stopped when TLC indicated the disappearance of the starting material and the presence of a ninhydrin-positive main spot (2 h). The mixture was filtered through a PTFE syringe filter (0.2 mm, 13 mm) and washed with a mixture of *t*-BuOH and H_2O (1/1, v/v, 2 mL), and the solvents were concentrated in vacuo. The residue was dissolved in distilled water (1.5 mL), and palladium hydroxide on carbon ($Pd(OH)_2$, Degussa type, 20%, 1.5 times the weight of starting material) was added. The resulting mixture was placed under an atmosphere of hydrogen, and after 12 h, TLC

(EtOAc/pyridine/water/AcOH 4/5/3/1, v/v/v/v) indicated the completion of the reaction. The mixture was filtered through a PTFE syringe filter, and the residue was washed with H_2O (2 mL). The filtrate was concentrated in vacuo, the residue was passed through a short column of Biorad 50 × 8 Na^+ resin (0.6 × 2.5 cm) using H_2O as the eluent, and the appropriate fractions were freeze dried to provide the final product.

General Procedure for Δ4-5 Elimination. To a solution of the starting material in DMF (1.0 mL for 0.02 mmol) was added DBU (1.1 equiv.). The reaction mixture was stirred at room temperature until TLC indicated completion (~2 h). The reaction was quenched by careful addition of AcOH. The mixture was concentrated in vacuo (water bath temperature ~ 20 °C), and the residue was applied to a P-2 Biogel column. The appropriate fractions were concentrated in vacuo (water bath temperature ~ 20 °C), and the residue was passed through a column of Biorad 50 × 8 Na^+ resin (0.6 × 5 cm) using H_2O as eluent, providing product.

General Procedure for Saponification of Methyl Esters. To a solution of the starting material in H_2O (0.02 M) was added 1 M NaOH until pH 9. The reaction mixture was left stirring for 8 h at room temperature. The mixture was then brought to pH 8-8.5 by careful addition of AcOH, and the mixture was concentrated in vacuo (water bath temperature ~ 20 °C). The residue was vortexed with water and applied to a RP-18 column (10 times the weight of starting material), which was eluted with a stepwise gradient of H_2O and CH_3OH (from 90/10 to 70/30, v/v). The appropriate fractions were concentrated in vacuo (water bath temperature ~ 20 °C), and the residue was passed through a column of Biorad 50 × 8 Na^+ resin (0.6 × 5 cm) using CH_3OH as eluent, providing product.

General Procedure for One Step Δ4-5 Elimination and Methyl Ester Hydrolysis. To a solution of the starting material in H_2O (1.0 mL for 0.02 mmol) was added NaOH (1 M, till pH ~ 9). The

reaction mixture was stirred at room temperature overnight until completion. The mixture was concentrated in vacuo (water bath temperature ~ 20 °C), and the residue was applied to a P-2 Biogel column. The appropriate fractions were concentrated in vacuo (water bath temperature ~ 20 °C), and the residue was passed through a column of Biorad 50 × 8 Na^+ resin (0.6 × 5 cm) using H_2O as eluent, providing product.

General Procedure for Selective *N*-Sulfation. To the solution of starting material in CH_3OH (1 mL for 0.006 mmol) were added triethylamine (0.3 mL) and 0.1 M NaOH (2 equiv. per NH_2) and Sulfur trioxide pyridine complex (20 equiv. per NH_2) at 0 °C. The progress of the reaction was monitored by TLC (silica gel TLC, EtOAc/pyridine/water/AcOH, 6/5/3/1, v/v/v/v). After stirring for an additional 8 h, the reaction mixture was co-evaporated with water (water bath temperature ~ 20 °C) and the residue passed through a short column of Biorad 50 × 8 Na^+ resin (0.6 × 5 cm) using CH_3OH and H_2O (90/10, v/v) as eluent. Appropriate fractions were concentrated in vacuo, and the residue was vortexed with water and applied to small RP-18 silica gel column (20 times the weight of starting material), which was then eluted with a stepwise gradient of H_2O and CH_3OH (90/10 to 40/60, v/v). The appropriate fractions were concentrated in vacuo to provide *N*-sulfated product. The residue was again passed through a short column of Biorad 50 × 8 Na^+ resin (0.6 × 5 cm) using CH_3OH and H_2O (90/10, v/v) as eluent.

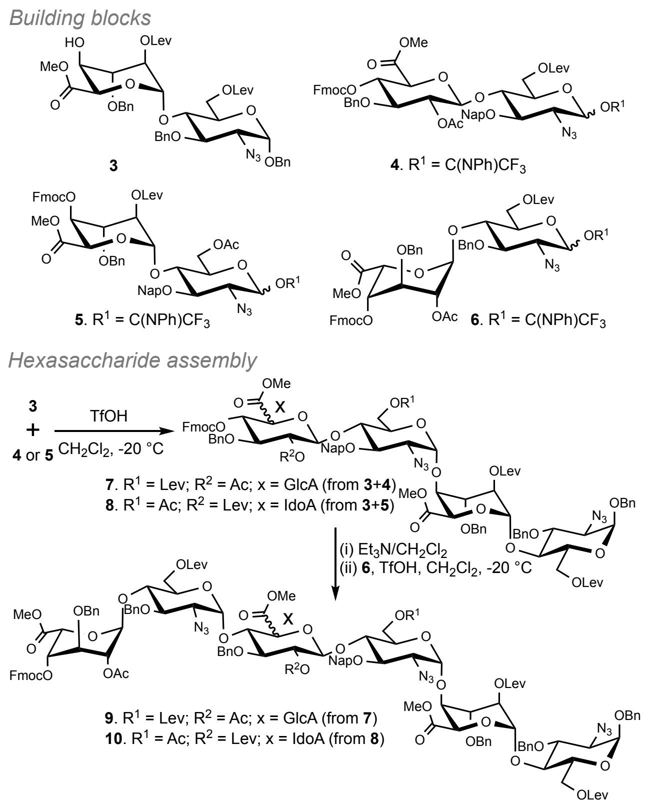

Scheme 1. Disaccharide building blocks for modular synthesis and glycosylation assembly of protected hexasaccharides.

Experimental Section

Benzyl-(methyl-2-*O*-levulinoyl-3-*O*-benzyl-α-L-idopyranosyluronate)]-(1→4)-*O*-2-azido-3-*O*-benzyl-2-deoxy-6-*O*-levulinoyl-α-D-glucopyranoside (3)

Compound **3** was prepared following the general procedure of glycosylation, benzylidene opening, oxidation and esterification. The structural characterization agrees with literature reports.[4]

^{1}H NMR (600 MHz, CDCl$_3$): δ 7.60 – 7.22 (m, 15H, Ar*H*), 5.05 – 5.02 (m, 2H, H-1^A, H-1^B), 4.96 (d, *J* = 2.3 Hz, 1H, H-2^B), 4.95 – 4.93 (m, 1H, H-5^B), 4.85 – 4.76 (m, 4H, 2 x CH$_2$-Bn), 4.75 – 4.69 (m, 3H, CH$_2$-Bn and H-4^B), 4.49 (dd, *J* = 12.6, 1.7 Hz, 1H, H-6A^A), 4.22 (dd, *J* = 12.5, 2.9 Hz, 1H, H-6B^A), 4.08 – 4.05 (m, 1H, H-3^B), 4.02 – 3.94 (m, 2H, H-4^A and H-5^A), 3.90 (dd, *J* = 10.3,

7.9 Hz, 1H, H-3^{A}), 3.48 (dd, *J* = 10.1, 3.6 Hz, 1H, H-2^{A}), 3.04 (s, 3H, COOC*H*$_3$), 2.85 – 2.57 (m, 8H, 4 x C*H*$_2$-Lev), 2.19 and 2.18 (2 x s, 2 x 3H, 2 x COC*H*$_3$).

^{13}C NMR (150 MHz, CDCl$_3$): δ 128.4 – 125.6 (Multiple Ar*C*), 97.3, 96.7, 78.5, 74.6, 74.6, 74.4, 73.6, 73.4, 73.1, 73.0, 69.9, 69.9, 69.4, 66.6, 66.5, 63.7, 62.3, 62.3, 51.7, 38.2, 37.9, 37.7, 29.6, 29.6, 28.1 and 28.0.

HRMS (MALDI-ToF) m/z: [M+Na-N$_2$]$^+$ Calcd. for $C_{44}H_{51}NNaO_{15}$ 856.31; Found 856.36.

Dimethylthexylsilyl-*O*-(methyl-2-*O*-acetyl-3-*O*-benzyl-4-*O*-[9-fluorenylmethyloxycarbonyl]-β-D-glucopyranoside)-(1→4)-*O*-2-azido-2-deoxy-3-*O*-napthylmethyl-6-*O*-levulinoyl-β-D-glucopyranoside (4a):

Compound **4a** was prepared following the general procedure of glycosylation, benzylidene opening, oxidation, esterification and Fmoc protection. The structural characterization agrees with literature reports.[1-4]

^{1}H NMR (500 MHz, CDCl$_3$): δ 8.00 – 7.14 (m, 20H, *Ar*H), 5.27 (d, *J* = 11.8 Hz, 1H, C*H*H-Nap), 5.14 (ddd, *J* = 17.5, 9.7, 8.6 Hz, 2H, H-2^{B} and H-4^{B}), 4.96 (d, J = 11.8 Hz, 1H, CH*H*-Nap), 4.79 (d, *J* = 8.0 Hz, 1H, H-1^{B}), 4.71 (d, J = 11.7 Hz, 1H, CHHBn), 4.60 (d, *J* = 11.7 Hz, 1H, C*H*H-Bn), 4.50 (d, *J* = 7.7 Hz, 1H, H-1^{A}), 4.43 (dd, *J* = 10.5, 7.1 Hz, 1H, C*H*H-Fmoc), 4.38 – 4.29 (m, 2H, H-6A^{A} and CH*H*-Fmoc), 4.29 – 4.11 (m, 3H, H6B^{A}, C*H*-Fmoc and H-5^{B}), 3.95 (t, *J* = 9.3 Hz, 1H, H-3^{B}), 3.85 (dd, *J* = 9.8, 8.5 Hz, 1H, H-4^{A}), 3.52 – 3.41 (m, 2H, H-5^{A} and H-3^{A}), 3.40 – 3.32 (m, 4H, COOC*H$_3$* and H-2^{A}), 2.89 (m, 1H, C*H*H-Lev), 2.78 – 2.61 (m, 2H, C*H$_2$*-Lev), 2.55 (m, 1H, CH*H*-Lev), 2.23 and 2.03 (2 x s, 2 x 3H, 2 x COC*H$_3$*), 1.73 – 1.65 (m, 1H, C*H*(CH$_3$)$_2$), 1.01 – 0.81 (m, 12H, CH(C*H$_3$*)$_2$ and C(C*H$_3$*)$_2$) and 0.20 (d, J = 4.4 Hz, 6H, Si(C*H$_3$*)$_2$).

^{13}C NMR (125 MHz, $CDCl_3$): δ 128.0 – 120.1 (multiple *Ar*C), 101.0, 96.9, 80.7, 79.5, 78.5,75.3, 75.2, 75.2, 75.2, 74.5, 74.5, 74.5, 74.5, 72.7, 72.6, 72.5, 70.4, 70.3, 68.7, 62.5, 62.5, 52.7, 46.7, 38.0, 38.0, 37.9, 34.0, 30.0, 29.9, 27.9, 27.9, 20.9, 20.0, 19.2, 16.2, 16.5 and -2.5. ‡Carbon assignments were obtained from HSQC experiment.

HRMS (MALDI-ToF) m/z: $[M+Na-N_2]^+$ Calcd. for $C_{61}H_{71}NNaO_{16}Si$ 1124.44; Found 1124.26. Compound **4a** was subjected to TDS-removal and *N*-phenylimidate installation to obtain donor **4**.

Dimethylthexylsilyl-*O*-(methyl-2-*O*-levulinoyl-3-*O*-benzyl-4-*O*-[9-fluorenylmethyloxycarbonyl]-α-L-idopyranoside)-(1→4)-*O*-2-azido-2-deoxy-3-*O*-napthylmethyl-6-*O*-acetyl-β-D-glucopyranoside (5a)

Compound **5a** was prepared following the general procedure of glycosylation, benzylidene opening, oxidation, esterification and Fmoc protection. The structural characterization agrees with literature reports.[1-4]

^{1}H NMR (500 MHz, $CDCl_3$): δ 7.86 – 7.22 (m, 20H, *Ar*H), 5.12 (s, 1H, H-1^B), 5.06 – 4.95 (m, 2H, H-5^B and H-4^B), 4.93 – 4.82 (m, 2H, H-2^B and C*H*H-Nap), 4.82 – 4.69 (m, 3H, CH*H*-Nap and C*H$_2$*-Bn), 4.61 – 4.49 (m, 2H, H-1^A and C*H*H-Fmoc), 4.45 (dd, J = 10.5, 7.3 Hz, 1H, H-6A^A), 4.39 – 4.31 (m, 1H, H-6B^A), 4.25 – 4.09 (m, 2H, C*H*-Fmoc and CH*H*-Fmoc), 3.97 – 3.86 (m, 2H, H-4^A and H-3^B), 3.56 – 3.46 (m, 1H, H-5^A), 3.46 – 3.38 (m, 1H, H-2^A), 3.35 – 3.21 (m, 1H, H-3^A), 3.02 (s, 3H, $COOCH_3$), 2.74 – 2.61 (m, 1H, C*H*H-Lev), 2.58 – 2.34 (m, 3H, CH*H*-Lev and CH_2-Lev), 2.09 and 2.05 (2 x s, 2 x 3H, 2 x $COCH_3$), 1.73 – 1.65 (m, 1H, $CH(CH_3)_2$), 0.94 – 0.85 (m, 12H, $CH(CH_3)_2$ and $C(CH_3)_2$) and 0.21 – 0.17 (m, 6H, $Si(CH_3)_2$).

^{13}C NMR (125 MHz, $CDCl_3$): δ 128.2 – 120.1 (multiple *Ar*C), 97.4, 97.1, 81.0, 74.9, 74.9, 74.5, 73.3, 72.9, 72.7, 71.2, 70.1, 70.1, 68.9, 67.1, 66.5, 62.5, 62.3, 51.7, 46.6, 37.4, 37.4, 33.9, 29.5, 28.1, 28.1, 21.8, 20.9, 18.3 and -2.3. ‡Carbon assignments were obtained from HSQC experiment.

HRMS (MALDI-ToF) m/z: $[M+Na-N_2]^+$ Calcd. for $C_{61}H_{71}NNaO_{16}Si$ 1124.44; Found 1124.26.

Compound **5a** was subjected to TDS-removal and *N*-phenylimidate installation to obtain donor **5**.

Dimethylthexylsilyl-*O*-(methyl-2-*O*-acetyl-3-*O*-benzyl-4-*O*-[9-fluorenylmethyloxy-carbonyl]-α-L-idopyranoside)-(1→4)-*O*-2-azido-2-deoxy-3-*O*-benzyl-6-*O*-levulinoyl-β-D-glucopyranoside (6a)

Compound **6a** was prepared following the general procedure of glycosylation, benzylidene opening, oxidation, esterification and Fmoc protection. The structural characterization agrees with literature reports.[1-4]

^{1}H NMR (300 MHz, $CDCl_3$): δ 7.78 – 7.14 (m, 18H, *Ar*H), 5.15 (s, 1H, H-1^B), 5.05 – 4.96 (m, 2H, H-4^B and H-5^B), 4.89 (t, J = 2.6 Hz, 1H, H-2^B), 4.90 – 4.52 (m, 4H, 2 x CH_2-Bn, C*H*H-Fmoc and H-1^A), 4.50 – 4.27 (m, 2H, H-6A^A and H-6B^A), 4.24 – 4.10 (m, 2H, C*H*-Fmoc and CH*H*-Fmoc), 3.94 – 3.82 (m, 2H, H-4^A and H-3^B), 3.52 – 3.49 (m, 1H, H-5^A), 3.48 (s, 3H, COOCH_3), 3.40 – 3.23 (m, 2H, H-2^A and H-3^A), 2.91 – 2.56 (m, 4H, 2 x CH_2 -Lev), 2.19 and 2.03 (2 x s, 2 x 3H, 2 x COCH_3), 1.74 – 1.64 (m, 1H, C*H*$(CH_3)_2$), 0.93 – 0.89 (m, 12H, $CH(CH_3)_2$ and C(CH_3)$_2$) and 0.22 (d, J = 4.3 Hz, 6H, Si(CH_3)$_2$).

^{13}C NMR (75 MHz, $CDCl_3$): δ 206.6, 172.1, 170.0, 168.3, 154.3, 143.2, 143.0, 141.3, 141.3, 137.9, 137.2, 129.1, 128.5, 128.5, 128.2, 128.2, 128.2, 128.1, 128.1, 128.1, 128.0, 128.0, 127.9, 127.8, 127.5, 127.4, 127.2, 127.1, 125.3, 125.1, 125.0, 120.1, 120.0, 97.3, 97.1, 80.9, 74.8, 74.2,

73.2, 72.9, 72.8, 71.2, 70.2, 69.0, 67.1, 66.6, 62.5, 52.2, 46.6, 37.9, 37.9, 34.0, 29.7, 28.1, 24.8, 20.9, 20.0, 20.0, 19.9, 18.5, 18.4, -2.1 and -3.3.

HRMS (MALDI-ToF) m/z: $[M+K]^+$ Calcd. for $C_{57}H_{69}N_3KO_{16}Si$ 1118.44; Found 1118.68.

Compound **6a** was subjected to TDS-removal and N-phenylimidate installation to obtain donor **6**.

Benzyl-*O*-[(methyl-2-*O*-acetyl-3-*O*-benzyl-4-*O*-(9-fluorenylmethoxycarbonyl)-β-D-glucopyranosyluronate)-(1→4)-*O*-(2-azido-3-*O*-2-naphthylmethyl-2-deoxy-6-*O*-levulinoyl-α-D-glucopyranoside)-(1→4)-*O*-(methyl-2-*O*-levulinoyl-3-*O*-benzyl-α-L-idopyranosyluronate)]-(1→4)-*O*-2-azido-3-*O*-benzyl-2-deoxy-6-*O*-levulinoyl-α-D-glucopyranoside (7)

Disaccharide donor **4** (200 mg, 0.172 mmol) and acceptor **3** (100.0 mg, 0.115 mmol) were coupled according to the general procedure for glycosylation to give tetrasaccharide **7** (100 mg, 47%) as white amorphous solid.

^{1}H NMR (500 MHz, CDCl$_3$): δ 7.94 – 7.72 (m, 8H, *Ar*H), 7.63 – 7.54 (m, 4H, *Ar*H), 7.50 – 7.17 (m, 38H, *Ar*H), 5.41 (d, *J* = 11.5 Hz, 1H, CH*H* of Nap), 5.25 (d, *J* = 4.2 Hz, 1H, $H1^D$), 5.20 – 5.09 (m, 2H, $H2^D$, $H4^D$), 5.06 (t, *J* = 3.9 Hz, 1H, $H1^C$), 5.00 – 4.92 (m, 2H, $H1^A$, $H2^B$), 4.87 – 4.74 (m, 3H, CH*H* of Nap, $H1^D$, CH*H* of Bn), 4.73 – 4.65 (m, 4H, 4 × CH*H* of Bn), 4.64 – 4.57 (m, 4H, $H5^B$ 3 × CH*H* of Bn), 4.48 – 4.17 (m, 8H, $H6ab^A$, $H6ab^C$, C*H₂* of Fmoc, $H5^D$, C*H* of Fmoc), 4.07 – 3.84 (m, 8H, $H3^D$, $H4^B$, $H3^B$, $H4^A$, $H5^A$, $H3^C$, $H4^C$, $H5^C$), 3.79 – 3.69 (m, 1H, $H3^C$), 3.56 – 3.48 (m, 3H, CO_2CH_3), 3.43 – 3.37 (m, $H2^A$, 1H), 3.36 – 3.29 (m, 4H, $H2^C$, CO_2CH_3), 2.98 – 2.42 (m, 12H, 6 × CH_2 of Lev), 2.27 –2.01 (4s, 12H, 3 × CH_3 of Lev, 1 × CH_3 of Ac).

^{13}C NMR (126 MHz, CDCl$_3$): δ 128.11– 120.05 (multiple *Ar*C), 100.65, 97.87, 97.28, 96.51, 79.40, 78.10, 77.86, 77.49, 75.47, 75.39, 75.23, 74.46, 74.46, 74.13, 72.85, 72.57, 72.39, 70.32,

70.31, 69.98, 69.72, 69.56, 69.42, 69.21, 63.29, 62.71, 62.32, 62.05, 61.90, 52.58, 52.19, 46.65, 38.17, 37.82, , 29.89, 29.87, 29.86, 29.74, 27.98, 20.76 and 20.64. ‡Carbon assignments were obtained from HSQC experiment.

HRMS (MALDI-ToF) m/z: $[M+Na]^+$ Calcd. for $C_{97}H_{102}N_6O_{30}Na$ 1854.8848; Found 1854.1488.

Benzyl-*O*-[(methyl-2-*O*-levulinoyl-3-*O*-benzyl-4-*O*-(9-fluorenylmethoxycarbonyl)-α-L-idopyranosyluronate)-(1→4)-*O*-(2-azido-3-*O*-2-naphthylmethyl-2-deoxy-6-*O*-acetyl-α-D-glucopyranoside)-(1→4)-*O*-(methyl-2-*O*-levulinoyl-3-*O*-benzyl-α-L-idopyranosyluronate)]-(1→4)-*O*-2-azido-3-*O*-benzyl-2-deoxy-6-*O*-levulinoyl-α-D-glucopyranoside (8)

Disaccharide donor **5** (150 mg, 0.129 mmol) and acceptor **3** (68 mg, 0.079 mmol) was coupled according to the general procedure for glycosylation to give tetrasaccharide **8** (70 mg, 48%) as white amorphous solid.

^{1}H NMR (500 MHz, CDCl$_3$): δ 7.88 – 7.66 (m, 9H, *Ar*H), 7.57 – 7.17 (m, 43H, *Ar*H), 5.23 (d, *J* = 3.6 Hz, 1H, $H1^B$), 5.14 (d, *J* = 2.8 Hz, 1H, $H1^D$), 5.10 (d, *J* = 3.6 Hz, 1H, $H1^C$), 4.99 – 4.94 (m, 3H, $H1^A$, $H2^B$, $H4^D$), 4.93 – 4.86 (m, 3H, $H2^D$, $H5^D$, CH*H* of Nap), 4.86 – 4.64 (m, 9H, $H5^B$, CH*H* of Nap, 7 × CH*H* of Bn), 4.59 (d, *J* = 12.0 Hz, 1H, CH*H* of Bn), 4.49 – 4.41 (m, 2H, $H6a^C$, $H6a^A$, CH*H* of Fmoc), 4.41 – 4.32 (m, 1H, CH*H* of Fmoc), 4.30 – 4.15 (m, 3H, $H6b^C$, $H6b^A$, C*H* of Fmoc), 4.07 – 4.02 (t, 1H, $H4^B$), 4.02 – 3.94 (m, 4H, $H5^C$, $H3^B$, $H4^C$, $H4^A$), 3.94 – 3.83 (m, 3H, $H3^C$, $H3^D$, $H5^A$), 3.74 – 3.67 (m, 1H, $H3^A$), 3.49 (s, 3H, CO_2CH_3), 3.38 (dd, *J* = 10.1, 4.0 Hz, 2H, $H2^A$, $H2^C$), 3.04 (s, 3H, CO_2CH_3), 2.83 – 2.38 (m, 12H, 6 × CH_2 of Lev), 2.21 – 1.98 (4s, 12H, 3 × CH_3 of Lev, 1 × CH_3 of Ac).

^{13}C NMR (126 MHz, CDCl$_3$): δ 128.15-120.02 (multiple *Ar*C), 97.74, 97.42, 96.70, 96.55, 78.49, 78.31, 75.41, 74.81, 74.80, 74.72, 74.69, 73.79, 73.43, 73.29, 73.25, 71.63, 70.96, 70.91, 70.23,

70.20, 69.84, 69.80, 69.67, 69.29, 69.29, 67.61, 63.39, 62.29, 62.22, 62.10, 51.83, 51.82, 46.67, 37.86, 37.69, 29.91, 29.72, 29.47, 28.01, 27.91, 27.72 and 20.99. ‡Carbon assignments were obtained from HSQC experiment.

HRMS (MALDI-ToF) m/z: $[M+Na]^+$ Calcd. for $C_{97}H_{102}N_6O_{30}Na$ 1854.8848; Found 1854.3793.

Benzyl-*O*-[(methyl-2-*O*-acetyl-3-*O*-benzyl-4-*O*-(9-fluorenylmethoxycarbonyl)-α-L-idopyranosyluronate)-(1→4)-*O*-(2-azido-3-*O*-benzyl-2-deoxy-6-*O*-levulinoyl-α-D-glucopyranoside)-(1→4)-*O*-(methyl-2-*O*-acetyl-3-*O*-benzyl-β-D-glucopyranosyluronate)-(1→4)-*O*-(2-azido-3-*O*-2-naphthylmethyl-2-deoxy-6-*O*-levulinoyl-α-D-glucopyranoside)-(1→4)-*O*-(methyl-2-*O*-levulinoyl-3-*O*-benzyl-α-L-idopyranosyluronate)]-(1→4)-*O*-2-azido-3-*O*-benzyl-2-deoxy-6-*O*-levulinoyl-α-D-glucopyranoside (9)

Tetrasaccharide **7** was subjected to Fmoc cleavage according to the general procedure to obtain a glycosyl acceptor (50 mg, 57%). Tetrasaccharide acceptor (50 mg, 0.031 mmol) was coupled with the donor **6** (71 mg, 0.062 mmol) according to the general procedure to obtain hexasaccharide **9** (51 mg, 66%) as white amorphous solid.

^{1}H NMR (600 MHz, $CDCl_3$): δ 7.93 – 7.69 (m, 8H, *Ar*H), 7.59 – 7.09 (m, 42H, *Ar*H), 5.40 (d, *J* = 3.8 Hz, 1H, $H1^E$), 5.29 (d, *J* = 11.1 Hz, 1H, CH*H* of Nap), 5.23 (d, *J* = 4.3 Hz, 1H, $H1^B$), 5.16 – 5.07 (m, 2H, $H2^D$, $H1^F$), 5.06 – 5.02 (m, 1H, $H1^C$), 4.94 (dt, *J* = 14.8, 4.7 Hz, 3H, $H1^A$, $H2^B$, $H4^F$), 4.89 – 4.77 (m, 4H, $H2^F$, $H5^F$, CH*H* of Nap, CH*H* of Bn), 4.77 – 4.62 (m, 10H, 9 × CH*H* of Bn, $H1^D$), 4.62 – 4.54 (m, 3H, 2 × CH*H* of Bn, $H5^B$), 4.45 – 4.33 (m, 5H, C*H₂* of Fmoc, $H6a^A$, $H6a^C$, $H6a^E$), 4.29 – 4.15 (m, 5H, $H6b^E$, $H6b^C$, C*H* of Fmoc, $H4^D$, $H5^D$), 4.08 (dd, *J* = 12.6, 3.0 Hz, 1H, $H6b^A$), 4.02 – 3.80 (m, 10H, $H3^D$, $H4^B$, $H3^B$, $H3^F$, $H3^A$, $H4^A$, $H4^C$, $H4^E$, $H5^C$, $H5^E$), 3.70 – 3.63 (m, 2H, $H3^C$, $H3^E$), 3.62 – 3.55 (m, 1H, $H5^A$), 3.54 – 3.40 (2s, 6H, 2 × CO_2CH_3), 3.39 – 3.20 (m,

6H, H2[A], H2[C], H2[E], 1 × CO_2CH_3), 2.98 – 2.38 (m, 16H, 8 × CH_2 of Lev), 2.24 – 2.07 (m, 12H, 4 × CH_3 of Lev), 2.02 – 1.94 (m, 6H, 2 × CH_3 of Ac).

^{13}C NMR (151 MHz, $CDCl_3$): δ 128.34-120.07 (multiple *Ar*C), 100.76, 97.82, 97.41, 97.32, 97.18, 96.59, 81.91, 78.35, 77.93, 77.53, 75.91, 75.46, 75.14, 74.99, 74.78, 74.65, 74.35, 74.35, 74.11, 73.61, 73.60, 73.45, 72.96, 72.43, 71.66, 70.15, 70.07, 69.65, 69.57, 69.50, 69.41, 69.10, 69.03, 68.00, 67.34, 63.29, 62.95, 62.93, 62.28, 62.04, 61.80, 61.80, 61.75, 61.73, 52.33, 52.17, 52.12, 46.56, 38.13, 37.79, 29.78, 29.74, 29.65, 27.97, 27.91, 27.86, 27.82 and 20.74. ‡Carbon assignments were obtained from HSQC experiment.

HRMS (MALDI-ToF) m/z: $[M + Na]^+$ Calcd. for $C_{131}H_{141}N_9O_{43}Na$ 2550.9021; Found 2550.9600.

Benzyl-*O*-[(methyl-2-*O*-acetyl-3-*O*-benzyl-4-*O*-(9-fluorenylmethoxycarbonyl)-α-L-idopyranosyluronate)-(1→4)-*O*-(2-azido-3-*O*-benzyl-2-deoxy-6-*O*-levulinoyl-α-D-glucopyranoside)-(1→4)-*O*-(methyl-2-*O*-levulinoyl-3-*O*-benzyl-α-L-idopyranosyluronate)-(1→4)-*O*-(2-azido-3-*O*-2-naphthylmethyl-2-deoxy-6-*O*-acetyl-α-D-glucopyranoside)-(1→4)-*O*-(methyl-2-*O*-levulinoyl-3-*O*-benzyl-α-L-idopyranosyluronate)]-(1→4)-*O*-2-azido-3-*O*-benzyl-2-deoxy-6-*O*-levulinoyl-α-D-glucopyranoside (10)

Tetrasaccharide **8** was subjected to Fmoc cleavage according to the general procedure to obtain a glycosyl acceptor (52 mg, 84%). Tetrasaccharide acceptor (52 mg, 0.031 mmol) was coupled with the donor **6** (72 mg, 0.062 mmol) according to the general procedure to obtain hexasaccharide **10** (50 mg, 64%) as white amorphous solid. **^{1}H NMR (800 MHz, $CDCl_3$):** δ 7.86 – 7.67 (m, 7H, *Ar*H), 7.61 – 7.15 (m, 39H, *Ar*H), 5.23 (d, J = 3.9 Hz, 1H, H1[B]), 5.21 (d, J = 4.0 Hz, 1H, H1[D]), 5.09 (dd, J = 5.7, 3.5 Hz, 2H, H1[F], H1[E]), 5.01 (d, J = 3.6 Hz, 1H, H1[C]), 4.97 – 4.89 (m, 5H, H1[A],

$H2^B$, $H2^D$, $H4^F$, CH*H* of Nap), 4.86 – 4.82 (m, 2H, $H5^F$, CH*H* of Nap), 4.82 – 4.77 (m, 3H, $H2^F$, $H5^B$, CH*H* of Bn), 4.77 – 4.55 (m, 12H, 11 × CH*H* of Bn, $H5^D$), 4.45 – 4.39 (m, 3H, $H6a^C$, $H6a^A$, CH*H* of Fmoc), 4.36 (dd, *J* = 10.7, 7.3 Hz, 2H, $H6a^E$, CH*H* of Fmoc), 4.25 (ddd, *J* = 26.9, 12.3, 3.6 Hz, 2H, $H6b^C$, $H6b^A$), 4.20 (t, *J* = 7.4 Hz, 1H, C*H* of Fmoc), 4.08 – 4.03 (m, 2H, $H6b^E$, $H4^B$), 3.95 (tq, *J* = 10.0, 5.3 Hz, 5H, $H3^B$, $H3^D$, $H4^D$, $H4^E$, $H4^A$), 3.92 – 3.82 (m, 5H, $H5^A$, $H3^A$, $H5^E$, $H4^C$, $H3^F$), 3.70 (dt, *J* = 18.4, 9.9 Hz, 2H, $H3^E$, $H5^C$), 3.58 (t, *J* = 9.7 Hz, 1H, $H3^C$), 3.53 – 3.43 (2s, 6H, 2 × CO_2CH_3), 3.35 (ddd, *J* = 13.6, 10.2, 3.5 Hz, 2H, $H2^A$, $H2^E$), 3.31 – 3.25 (m, 1H, $H2^C$), 3.20 (S, 3H, CO_2CH_3), 2.84 – 2.47 (m, 16H, 8 × CH_2 of Lev), 2.20 – 1.91 (6s, 18H, 4 × CH_3 of Lev, 2 × CH_3 of Ac).

^{13}C NMR (201 MHz, $CDCl_3$): δ 128.19-120.05 (multiple *Ar*C), 97.83, 97.78, 97.19, 96.71, 96.64, 96.51, 78.29, 77.96, 77.93, 76.22, 75.50, 75.25, 74.84, 74.81, 74.74, 74.74, 74.69, 73.78, 73.51, 73.37, 73.33, 71.77, 71.70, 71.61, 70.26, 70.14, 69.72, 69.65, 69.58, 69.52, 69.48, 69.40, 69.35, 68.46, 67.39, 63.20, 63.16, 62.30, 62.08, 62.00, 61.87, 61.80, 52.13, 51.86, 51.50, 46.60, 37.88, 37.76, 37.68, 29.76, 29.67, 29.59, 29.53, 27.89, 27.89, 27.64, 20.85 and 20.77. ‡Carbon assignments were obtained from HSQC experiment.

HRMS (MALDI-ToF) m/z: $[M + Na]^+$ Calcd. for $C_{131}H_{141}N_9O_{43}Na$ 2550.9021; Found 2550.8838.

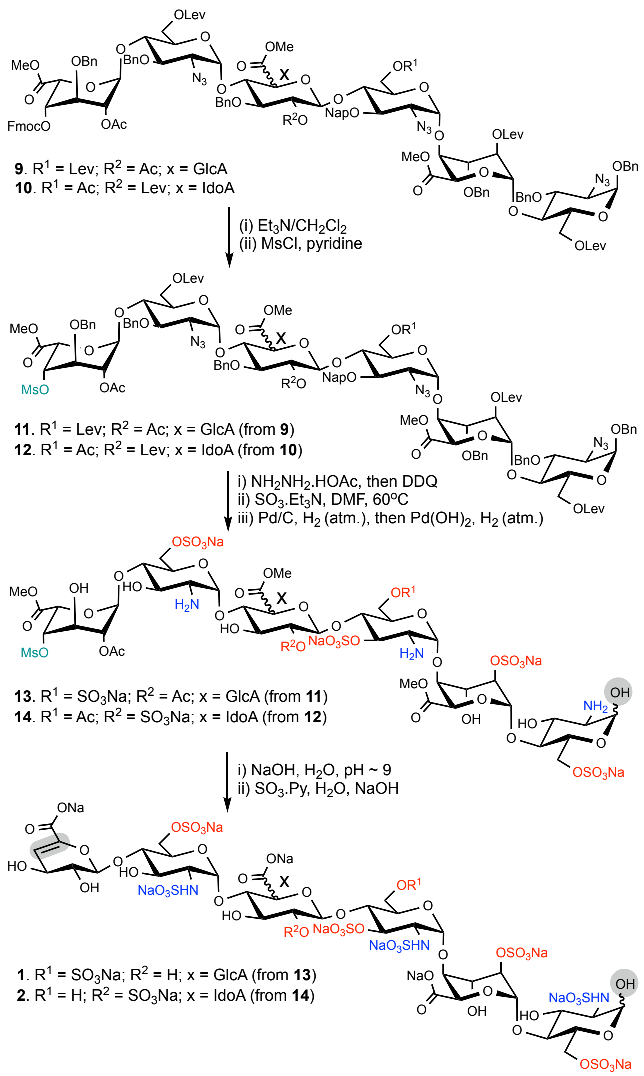

Scheme 2. Preparation of hexasaccharides **1** and **2**.

Benzyl-*O*-[(methyl-2-*O*-acetyl-3-*O*-benzyl-4-*O*-mesyl-α-L-idopyranosyluronate)-(1→4)-*O*-(2-azido-3-*O*-benzyl-2-deoxy-6-*O*-levulinoyl-α-D-glucopyranoside)-(1→4)-*O*-(methyl-2-*O*-acetyl-3-*O*-benzyl-β-D-glucopyranosyluronate)-(1→4)-*O*-(2-azido-3-*O*-2-naphthylmethyl-2-deoxy-6-*O*-levulinoyl-α-D-glucopyranoside)-(1→4)-*O*-(methyl-2-*O*-levulinoyl-3-*O*-benzyl-α-L-idopyranosyluronate)]-(1→4)-*O*-2-azido-3-*O*-benzyl-2-deoxy-6-*O*-levulinoyl-α-D-glucopyranoside (11)

Hexasaccharide **9** was subjected to Fmoc removal and mesylation according to general procedures to obtain hexasaccharide **11** (43 mg, 65%) as beige amorphous solid.

^{1}H NMR (500 MHz, CDCl$_3$): δ 7.96 – 7.74 (m, 5H, *Ar*H), 7.59 – 7.08 (m, 64H, *Ar*H), 5.43 (d, *J* = 3.9 Hz, 1H, H1^E), 5.34 – 5.28 (m, 1H, CH*H* of Nap), 5.24 (d, *J* = 4.2 Hz, 1H, H1^B), 5.16 (t, *J* = 8.8 Hz, 1H, H2^D), 5.06 (d, *J* = 4.0 Hz, 2H, H1^C, H1^F), 4.98 – 4.91 (m, 2H, H1^A, H2^B), 4.91 – 4.54 (m, 18H, H2^F, H4^F, H5^F, H1^D, H5^B, CH*H* of Nap, 6 × C*H$_2$* of Bn), 4.40 (t, *J* = 13.9 Hz, 3H, H6a^A, H6a^C, H6a^E), 4.32 – 4.16 (m, 4H, H6b^C, H6b^E, H4^D, H5^D), 4.15 – 3.80 (m, 11H, H6b^A, H3^F, H4^B, H3^B, H3^D, H3^A, H4^A, H4^C, H4^E, H5^C, H5^E), 3.68 (q, *J* = 10.1 Hz, 2H, H3^C, H3^E), 3.63 – 3.45 (m, 7H, H5^A, 2 × CO$_2$C*H$_3$*), 3.41 – 3.23 (m, 6H, H2^A, H2^C, H2^E, 1 × CO$_2$C*H$_3$*), 3.03 – 2.43 (19H, C*H$_3$* of Ms, 8 × C*H$_2$* of Lev), 2.26 – 1.97 (6s, 18H, 4 × C*H$_3$* of Lev, 2 × C*H$_3$* of Ac).

^{13}C NMR (126 MHz, CDCl$_3$): δ 128.42-123.98 (multiple *Ar*C), 100.82, 97.97, 97.45, 97.36, 96.58, 82.07, 78.25, 78.03, 75.82, 75.48, 75.30, 74.99, 74.83, 74.77, 74.51, 74.29, 74.23, 74.07, 73.92, 73.61, 73.22, 72.91, 72.69, 69.96, 69.71, 69.69, 69.66, 69.55, 69.06, 67.40, 67.06, 63.38, 63.23, 62.98, 62.40, 62.33, 62.07, 62.01, 61.86, 61.70, 52.35, 52.20, 38.85, 37.87, 29.80, 29.79, 29.70, 27.94, 27.74, 27.71, 20.83 and 20.78. ‡Carbon assignments were obtained from HSQC experiment.

HRMS (MALDI-ToF) m/z: [M + Na]$^+$ Calcd. for $C_{117}H_{133}N_9NaO_{43}S$ 2406.8116; Found 2406.0786.

Benzyl-*O*-[(methyl-2-*O*-acetyl-3-*O*-benzyl-4-*O*-mesyl-α-L-idopyranosyluronate)-(1→4)-*O*-(2-azido-3-*O*-benzyl-2-deoxy-6-*O*-levulinoyl-α-D-glucopyranoside)-(1→4)-*O*-(methyl-2-*O*-levulinoyl-3-*O*-benzyl-α-L-idopyranosyluronate)-(1→4)-*O*-(2-azido-3-*O*-2-naphthylmethyl-2-deoxy-6-*O*-acetyl-α-D-glucopyranoside)-(1→4)-*O*-(methyl-2-*O*-levulinoyl-3-*O*-benzyl-α-L-

idopyranosyluronate)]-(1→4)-*O*-2-azido-3-*O*-benzyl-2-deoxy-6-*O*-levulinoyl-α-D-glucopyranoside (12)

Hexasaccharide **10** was subjected to Fmoc removal and mesylation according to general procedures to obtain hexasaccharide **12** (33 mg, 59%) as beige amorphous solid.

^{1}H NMR (600 MHz, $CDCl_3$): δ 7.88 – 7.65 (m, 4H, *Ar*H), 7.52 – 7.02 (m, 33H, *Ar*H), 5.22 (t, *J* = 4.0 Hz, 1H, $H1^B$), 5.18 (t, *J* = 4.2 Hz, 1H, $H1^D$), 5.08 (t, *J* = 4.2 Hz, 1H, $H1^E$), 5.05 – 4.98 (m, 2H, $H1^F$, $H1^C$), 4.92 (ddd, *J* = 13.4, 9.6, 4.0 Hz, 4H, $H2^B$, $H2^D$, $H1^A$, CH*H* of Nap), 4.88 – 4.52 (m, 18H, CH*H* of Nap, $H5^F$, $H2^F$, $H4^F$, 6 × C*H_2* of Bn, $H5^B$, $H5^D$), 4.43 – 4.24 (m, 5H, $H6ab^A$, $H6ab^E$, $H6a^C$), 4.07 – 3.80 (m, 12H, $H6b^C$, $H4^B$, $H3^F$, $H3^B$, $H3^D$, $H4^D$, $H4^E$, $H5^E$, $H4^C$, $H3^A$, $H4^A$, $H5^A$), 3.67 (dt, *J* = 34.4, 9.1 Hz, 2H, $H3^E$, $H5^C$), 3.61 – 3.54 (m, 1H, $H3^C$), 3.52 – 3.42 (2s, 6H, 2 × CO_2CH_3), 3.38 – 3.25 (m, 3H, $H2^A$, $H2^C$, $H2^E$), 3.18 – 3.11 (m, 3H, CO_2CH_3), 2.88 (d, *J* = 3.8 Hz, 3H, C*H_3* of Ms), 2.82 – 2.45 (16H, 8 × C*H_2* of Lev), 2.20 – 1.96 (6s, 18H, 4 × C*H_3* of Lev, 2 × C*H_3* of Ac).

^{13}C NMR (151 MHz, $CDCl_3$): δ 128.36-125.84 (multiple *Ar*C), 97.85, 97.81, 71.57, 97.23, 96.96, 96.83, 96.52, 78.36, 78.00, 77.96, 75.44, 75.15, 74.88, 74.77, 74.77, 74.70, 74.59, 74.54, 74.18, 73.68, 73.54, 73.41, 71.69, 70.75, 69.71, 69.64, 69.58, 69.49, 69.33, 69.26, 69.23, 67.37, 67.24, 63.20, 63.17, 62.20, 62.18, 62.03, 61.81, 61.80, 52.08, 51.44, 38.25, 37.86, 37.77, 37.67, 29.70, 29.69, 29.56, 27.87, 27.79, 27.65, 20.84 and 20.78. ‡Carbon assignments were obtained from HSQC experiment.

HRMS (MALDI-ToF) m/z: $[M + Na]^+$ Calcd. for $C_{117}H_{133}N_9NaO_{43}S$ 2406.8116; Found 2407.0000.

Methyl-2-*O*-acetyl-4-*O*-mesyl-α-L-idopyranosyluronate-(1→4)-*O*-(2-amino-2-deoxy-6-*O*-sulfonate-α-D-glucopyranoside)-(1→4)-*O*-(methyl-2-*O*-acetyl-β-D-glucopyranosyluronate)-(1→4)-*O*-(2-amino-2-deoxy-3,6-disulfonate-α-D-glucopyranoside)-(1→4)-*O*-(methyl-2-*O*-sulfonate-α-L-idopyranosyluronate)-(1→4)-*O*-2-amino-2-deoxy-6-*O*-sulfonate-α-D-glucopyranoside (13)

Hexasaccharide **11** was subjected to Lev removal, Nap removal, *O*-sulfation, and hydrogenation according to general procedures to obtain hexasaccharide **13** (10mg, 21%) as white fluffy solid.

^{1}H NMR (600 MHz, D_2O): δ 5.34 (t, J = 4.2 Hz, 2H, H1^E, H1^C), 5.26 (d, J = 3.6 Hz, 1H, H1^A), 5.19 – 5.12 (m, 3H, H1^B, H5^B, H5^F), 5.00 (d, J = 8.3 Hz, 1H, H1^F), 4.89 (t, J = 3.3 Hz, 1H, H2^B), 4.79 – 4.63 (m, 3H, H2^D, H2^F, H1^D), 4.34 – 4.23 (m, 3H, H3^C, H4^D), 4.23 – 4.00 (m, 11H, H3^B, H3^F, H4^B, H4^F, H6ab^A, H6ab^C, H6ab^E, H4^C), 4.00 – 3.46 (m, 19H, H3^A, H3^E, H4^A, H4^E, H5^A, H5^E, H3^D, H5^D 3 × CO_2CH_3, H2^C, H5^C), 3.22 – 3.04 (m, 5H, H2^A, H2^E, CH_3 of Ms), 2.10 (d, J = 3.9 Hz, 3H, CH_3 of Ac), 2.04 – 1.97 (m, 3H, CH_3 of Ac).

^{13}C NMR (151 MHz, D_2O): δ 100.24, 99.40, 98.94, 96.84, 91.70, 89.22, 83.67, 82.54, 78.84, 77.41, 76.98, 76.18, 75.04, 74.30, 74.14, 73.33, 73.11, 72.90, 72.85, 70.11, 70.00, 69.32, 69.04, 68.84, 68.44, 68.36, 67.08, 66.68, 65.68, 65.57, 65.51, 65.44, 65.33, 65.19, 62.30, 54.38, 53.17, 53.16, 52.99, 37.70, 20.29 and 20.25. ‡Carbon assignments were obtained from HSQC experiment.

HRMS (ESI) m/z: $[M-6Na+4H]^{2-}$ Calcd. for $C_{44}H_{59}N_3O_{50}S_6$ 815.5642; Found 815.5554.

Methyl-2-*O*-acetyl-4-*O*-mesyl-α-L-idopyranosyluronate-(1→4)-*O*-(2-amino-2-deoxy-6-*O*-sulfonate-α-D-glucopyranoside)-(1→4)-*O*-(methyl-2-*O*-sulfonate-α-L-idopyranosyluronate)-(1→4)-*O*-(2-amino-3-*O*-sulfonate-2-deoxy-6-*O*-acetyl-α-D-glucopyranoside)-(1→4)-*O*-(methyl-2-*O*-sulfonate-α-L-idopyranosyluronate)]-(1→4)-*O*-2-amino-2-deoxy-6-*O*-sulfonate-α-D-glucopyranoside (14)

Hexasaccharide **12** was subjected to Lev removal, Nap removal, *O*-sulfation, and hydrogenation according to general procedures to obtain hexasaccharide **14** (8 mg, 28%) as white fluffy solid.

^{1}H NMR (500 MHz, D_2O): δ 5.42 (t, *J* = 1.8 Hz, 1H, H5^{D}), 5.30 – 5.20 (m, 3H, H1^{A}, H1^{C}, H1^{E}), 5.20 – 5.11 (m, 4H, H1^{B}, H1^{D}, H5^{B}, H5^{F}), 5.05 – 5.00 (m, 1H, H1^{F}), 4.93 (q, *J* = 4.0, 3.3 Hz, 2H, H2^{B}, H2^{D}), 4.74 (dd, *J* = 4.5, 2.4 Hz, 1H, H2^{F}), 4.59 (d, *J* = 12.3 Hz, 1H, H6a^{C}), 4.39 – 4.32 (m, 1H, H3^{C}), 4.30 – 3.95 (m, 11H, H6b^{C}, H6ab^{A}, H6ab^{E}, H3B, H4^{B}, H3^{D}, H4^{C}, H3^{F}, H4^{F}), 3.92 – 3.83 (m, 1H, H4^{C}), 3.83 – 3.50 (m, 16H, 3 × CO_2CH_3, H3^{A}, H3^{E}, H4^{A}, H4^{E}, H5^{A}, H5^{C}, H5^{E}), 3.41 – 3.32 (m, 1H, H2^{C}), 3.14 (m, 5H, C*H₃* of Ms, H2^{A}, H2^{E}), 2.07 – 2.00 (2s, 6H, 2 × CH_3 of Ac).

^{13}C NMR (126 MHz, D_2O): δ 99.58, 99.14, 98.90, 92.78, 92.43, 89.62, 77.17, 76.52, 76.16, 75.79, 72.60, 72.46, 70.63, 70.61, 69.81, 69.70, 69.70, 68.57, 66.93, 66.89, 66.68, 66.61, 66.60, 66.59, 66.57, 66.56, 65.51, 62.52, 62.00, 61.92, 54.60, 54.13, 53.65, 53.24, 52.94, 52.81, 52.78, 37.83 and 20.86. ‡Carbon assignments were obtained from HSQC experiment.

HRMS (ESI) m/z: $[M\text{-}6Na+4H]^{2-}$ Calcd. for $C_{44}H_{59}N_3O_{50}S_6$ 815.5642; Found 815.5554.

α-L-Threo-hex-4-deoxy-enopyranosyluronate-(1→4)-*O*-(2-sulfamino-2-deoxy-6-*O*-sulfonate-α-D-glucopyranoside)-(1→4)-*O*-(β-D-glucopyranosyluronate)-(1→4)-*O*-(2-sulfamino-2-deoxy-3,6-disulfonate-α-D-glucopyranoside)-(1→4)-*O*-(2-*O*-sulfonate-α-L-idopyranosyluronate)-(1→4)-*O*-2-sulfamino-2-deoxy-6-*O*-sulfonate-α-D-glucopyranoside (1)

Hexasaccharide **13** was subjected to elimination, saponification, de-*O*-acetylation, and *N*-sulfation according to general procedures to obtain the final hexasaccharide **1** (7 mg, 62%) as white fluffy solid.

^{1}H NMR (600 MHz, D_2O): δ 5.66 – 5.62 (m, 1H, H4^F), 5.47 (d, *J* = 3.8 Hz, 1H, H1^E), 5.39 (d, *J* = 5.3 Hz, 1H, H1^C), 5.33 – 5.23 (m, 1H, H1^A), 5.00 (dd, *J* = 13.2, 5.4 Hz, 2H, H1^B, H1^F), 4.60 – 4.41 (m, 3H, H5^B, H3^C, H1^D), 4.38 – 4.26 (m, 2H, H6a^A, H6a^E), 4.26 – 3.77 (m, 15H, H6b^A, H6b^E, H6ab^C, H2^B, H3^F, H3^B, H4^B, H4^C, H5^A, H5^D, H5^C, H5^E, H3^D H4^D), 3.76 – 3.42 (m, 5H, H2^F, H3^A, H3^E, H4^A, H4^E), 3.33 – 3.21 (m, 2H, H2^C, H2^D), 3.14 (ddd, *J* = 29.9, 10.5, 3.8 Hz, 2H, H2^A, H2^E).
^{13}C NMR (151 MHz, D_2O): δ 107.65, 100.94, 100.76, 99.67, 97.10, 95.75, 91.02, 77.71, 76.58, 76.51, 76.12, 75.95, 75.62, 72.69, 72.60, 70.57, 70.41, 70.35, 70.16, 70.03, 70.00, 69.34, 68.79, 66.22, 66.13, 65.83, 65.73, 65.72, 65.72, 57.78, 57.65 and 56.54. ‡Carbon assignments were obtained from HSQC experiment.
HRMS (ESI) m/z: $[M-7Na+5H]^{2-}$ Calcd. for $C_{86}H_{54}N_3NaO_{54}S_8$ 835.4623; Found 835.0736.

α-L-Threo-hex-4-deoxy-enopyranosyluronate-(1→4)-*O*-(2-sulfamino-2-deoxy-6-*O*-sulfonate-α-D-glucopyranoside)-(1→4)-*O*-(2-*O*-sulfonate-α-L-idopyranosyluronate)-(1→4)-*O*-(2-sulfamino-3-*O*-sulfonate-2-deoxy-α-D-glucopyranoside)-(1→4)-*O*-(2-*O*-sulfonate-α-L-idopyranosyluronate)]-(1→4)-*O*-2-sulfamino-2-deoxy-6-*O*-sulfonate-α-D-glucopyranoside (2)

Hexasaccharide **14** was subjected to elimination, saponification, de-*O*-acetylation, and *N*-sulfation according to general procedures to obtain the final hexasaccharide **2** (6 mg, 65%) as white fluffy solid.

^{1}H NMR (600 MHz, D_2O): δ 5.64 (d, *J* = 3.4 Hz, 1H, H4^F), 5.35 (s, 1H, H1^C), 5.30 (d, *J* = 3.6 Hz, 1H, H1^E), 5.26 – 5.14 (m, 1H, H1^A), 5.03 – 4.94 (m, 3H, H1^B, H1^D, H1^F), 4.86 – 4.80 (m, 1H, H5^D), 4.60 – 4.50 (m, 1H, H5^B), 4.35 – 4.28 (m, 1H, H6a^E), 4.28 – 3.90 (m, 12H, H6b^E, H6ab^A,

$H3^C$, $H5^C$, $H2^B$, $H2^D$, $H3^B$, $H3^D$, $H4^B$, $H4^D$, $H3^F$), 3.90 – 3.45 (m, 10H, $H3^A$, $H3^E$, $H4^C$, $H4^A$, $H4^E$, $H5^A$, $H5^E$, $H2^F$, $H6ab^C$), 3.35 – 3.26 (m, 1H, $H2^C$), 3.23 – 3.09 (m, 2H, $H2^A$, $H2^E$).

^{13}C NMR (151 MHz, D_2O): δ 107.68, 99.76, 98.79, 97.45, 96.32, 91.40, 90.96, 77.66, 77.54, 76.09, 75.15, 74.47, 73.12, 72.72, 70.65, 70.46, 70.37, 70.19, 70.14, 69.29, 68.87, 68.86, 68.12, 67.92, 66.46, 66.41, 65.76, 65.58, 62.81, 59.35, 57.85, 57.71 and 56.96. ‡Carbon assignments were obtained from HSQC experiment.

HRMS (ESI) m/z: $[M\text{-}7Na+4H]^{3-}$ Calcd. for $C_{86}H_{54}N_3NaO_{54}S_8$ 556.6391; Found 556.3758.

References

(1) Arungundram, S.; Al-Mafraji, K.; Asong, J.; Leach, F. E., III; Amster, I. J.; Venot, A.; Turnbull, J. E.; Boons, G. J. Modular synthesis of heparan sulfate oligosaccharides for structure-activity relationship studies. *J. Am. Chem. Soc.* **2009**, *131* (47), 17394-17405.

(2) Zong, C.; Venot, A.; Li, X.; Lu, W.; Xiao, W.; Wilkes, J. L.; Salanga, C. L.; Handel, T. M.; Wang, L.; Wolfert, M. A.; Boons, G. J. Heparan sulfate microarray reveals that heparan sulfate-protein binding exhibits different ligand requirements. *J. Am. Chem. Soc.* **2017**, *139* (28), 9534-9543.

(3) Chopra, P.; Joshi, A.; Wu, J.; Lu, W.; Yadavalli, T.; Wolfert, M. A.; Shukla, D.; Zaia, J.; Boons, G. J. 3-*O*-Sulfation of heparan sulfate modulates protein binding and lyase degradation. *Proc. Natl. Acad. Sci. U. S. A.* **2021**, *118* (3), e2012935118.

(4) Karlsson, R.; Chopra, P.; Joshi, A.; Yang, Z.; Vakhrushev, S. Y.; Clausen, T. M.; Painter, C. D.; Szekeres, G. P.; Chen, Y. H.; Sandoval, D. R.; Hansen, L.; Esko, J. D.; Pagel, K.; Dyer, D. P.; Turnbull, J. E.; Clausen, H.; Boons, G. J.; Miller, R. L. Dissecting structure-function of 3-O-sulfated heparin and engineered heparan sulfates. *Sci. Adv.* **2021**, *7* (52), eabl6026.

Spectral Data

Figure S1. ^{1}H NMR (600 MHz, $CDCl_3$) spectrum of compound **3**.

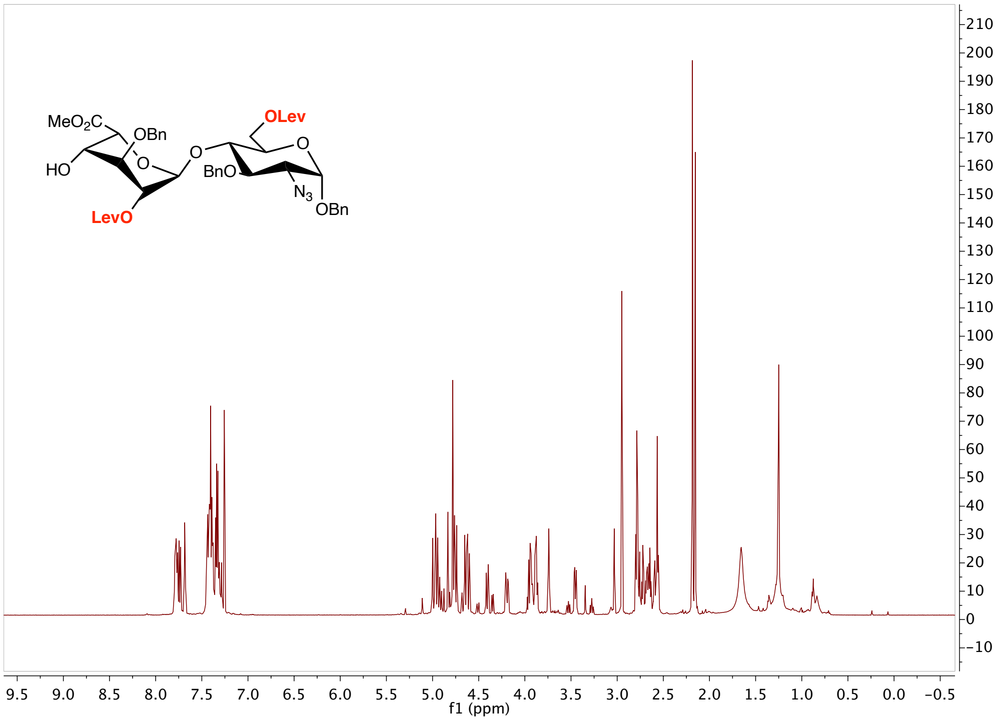

Figure S2. ^{1}H NMR (500 MHz, $CDCl_3$) spectrum of compound **4a**.

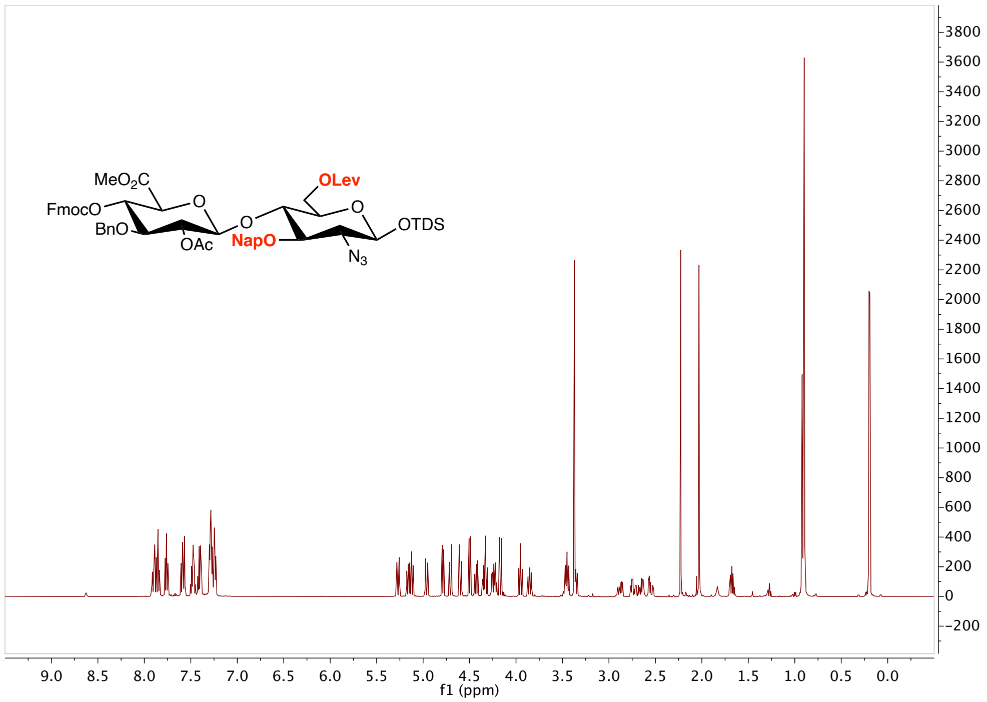

Figure S3. ^{1}H NMR (500 MHz, $CDCl_3$) spectrum of compound **5a**.

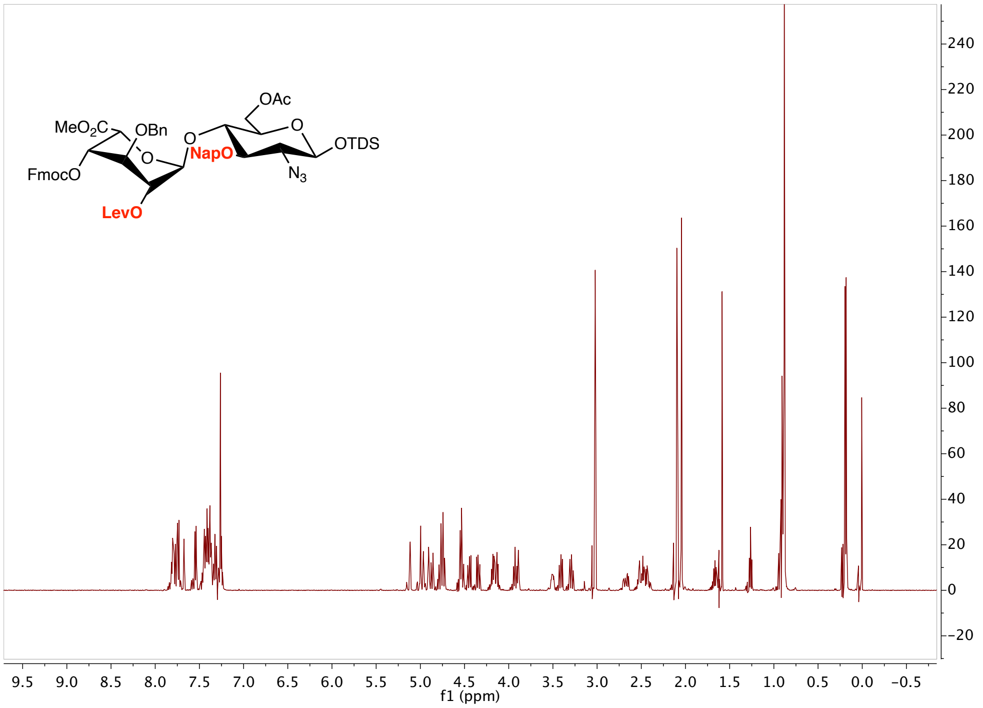

Figure S4. ^{1}H NMR (300 MHz, $CDCl_3$) spectrum of compound **6a**.

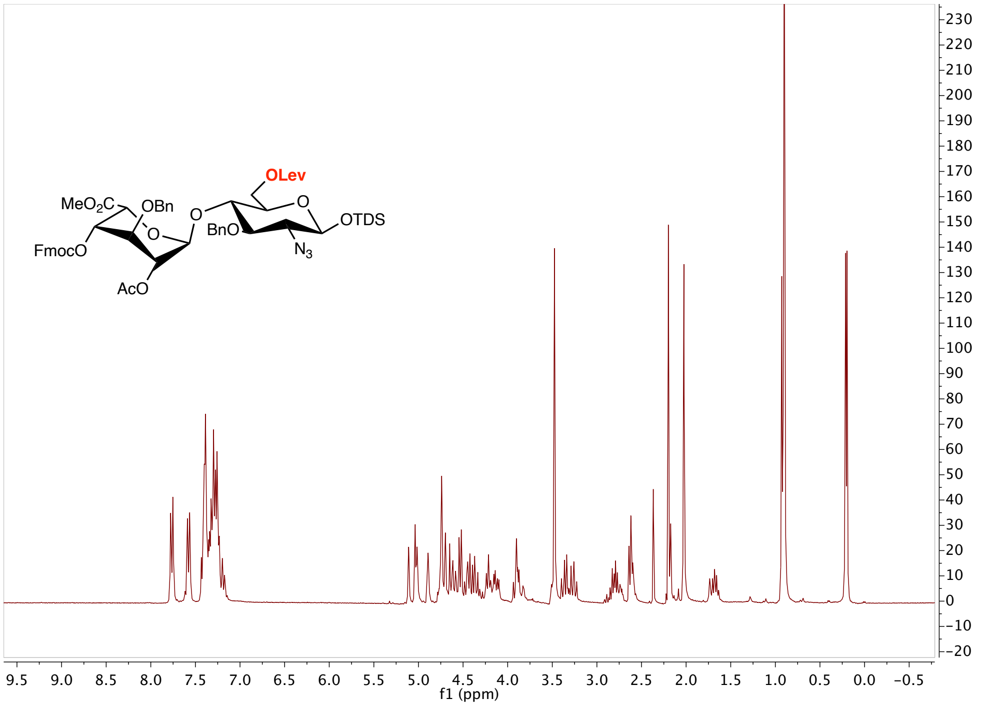

Figure S5. ^{1}H and HSQC NMR (500 MHz, $CDCl_3$) spectra of compound **7**.

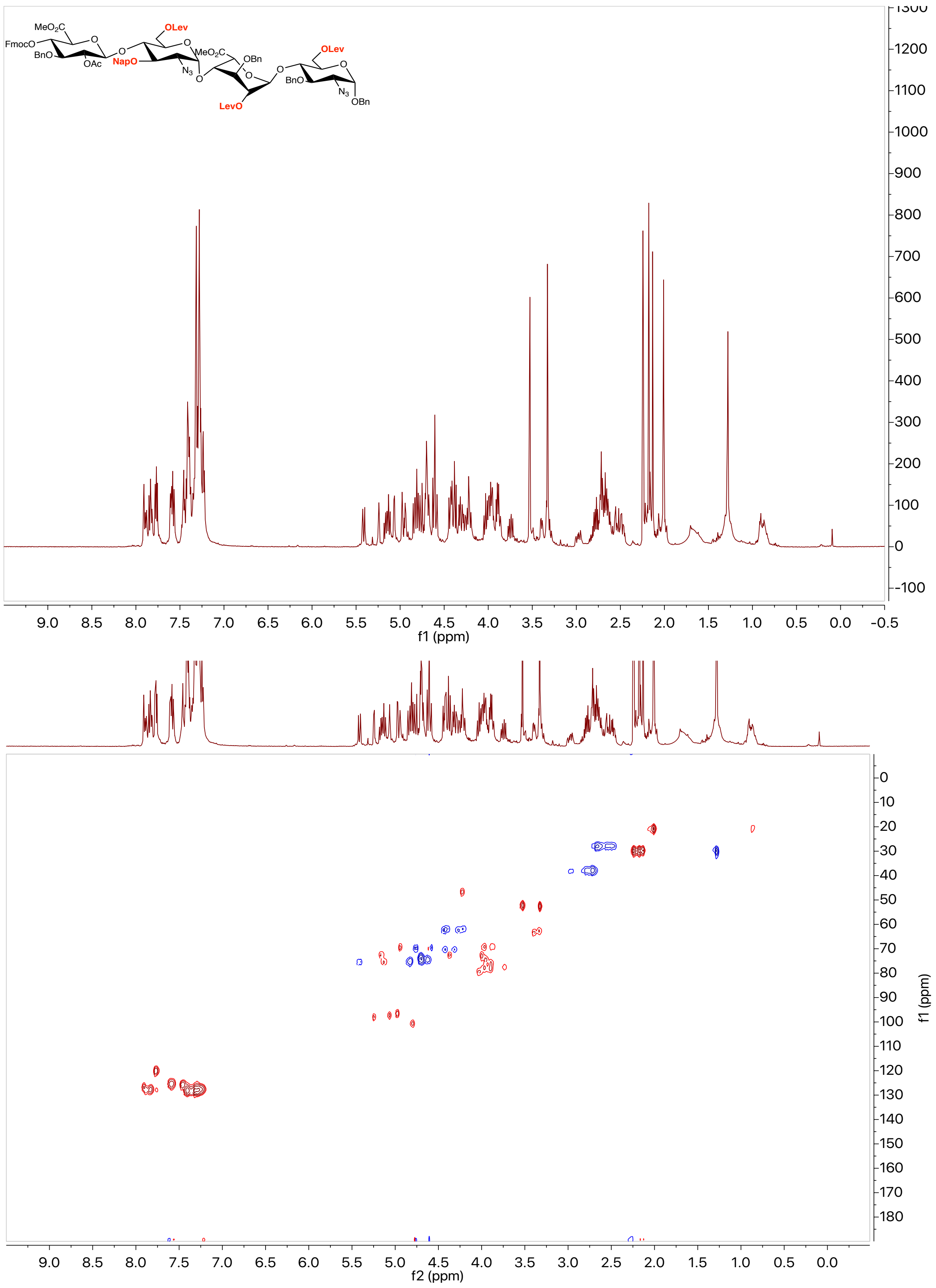

Figure S6. ^{1}H and HSQC NMR (500 MHz, $CDCl_3$) spectra of compound **8**.

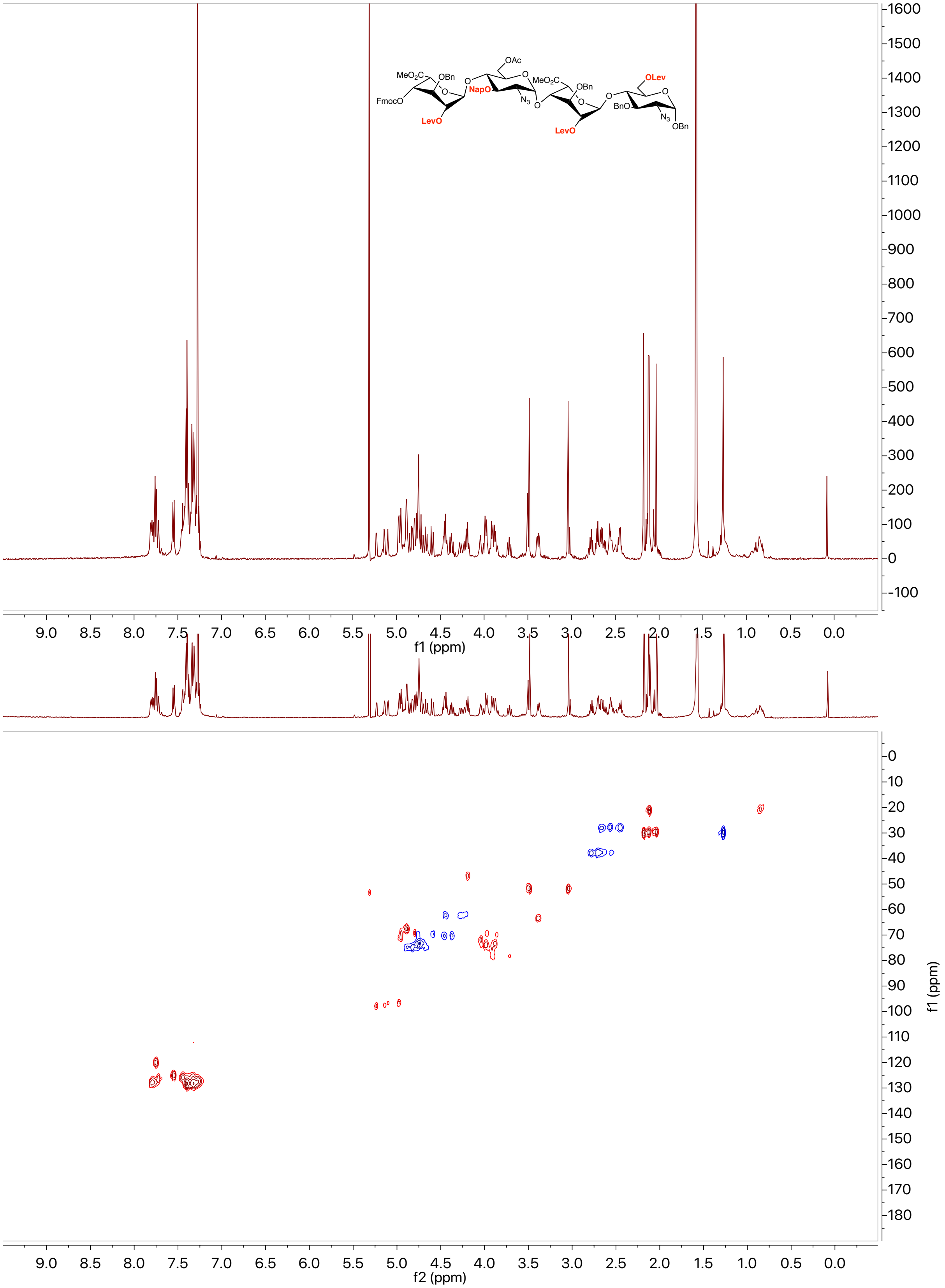

Figure S7. ^{1}H and HSQC NMR (600 MHz, $CDCl_3$) spectra of compound **9**.

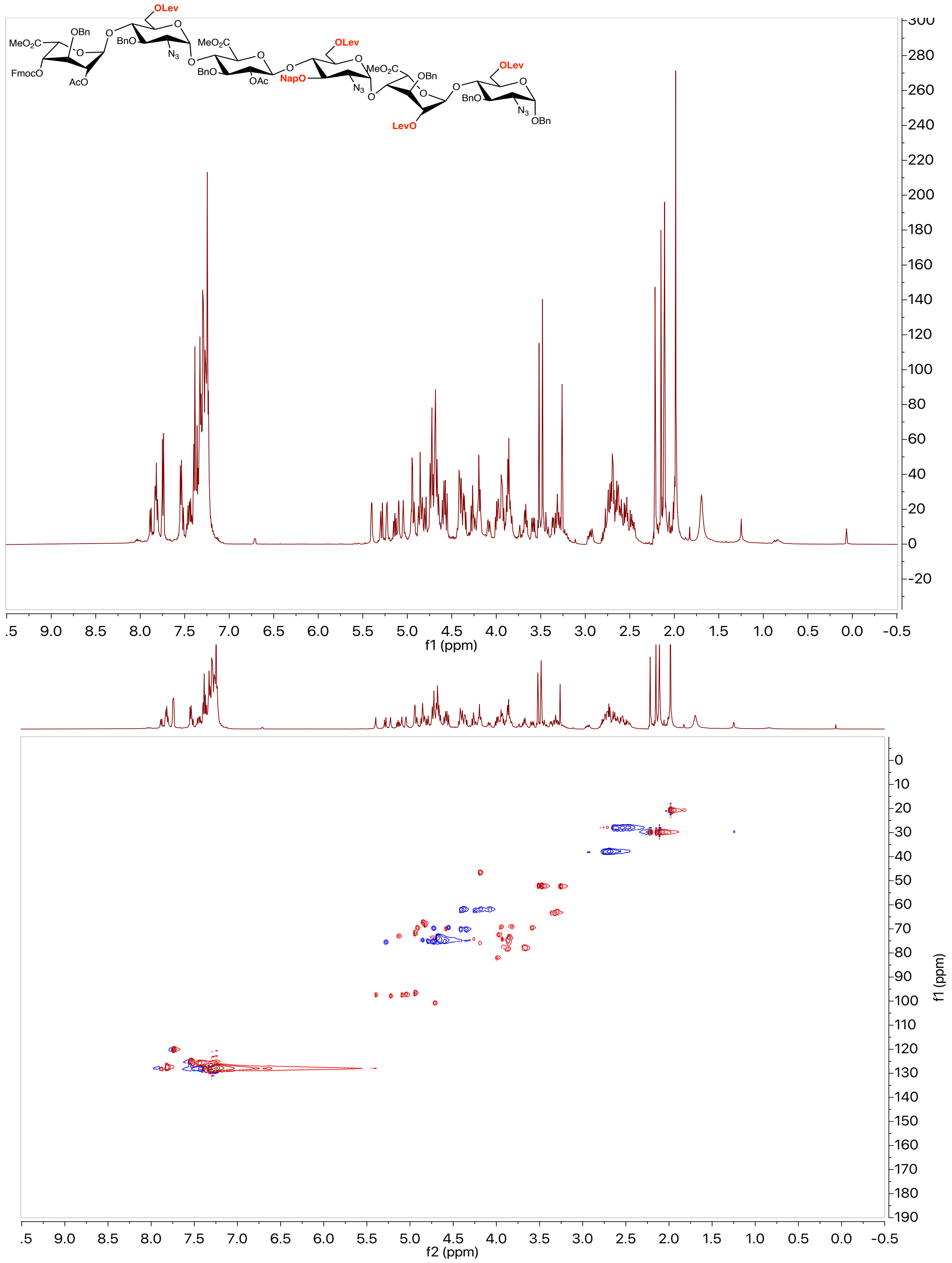

Figure S8. ^{1}H and HSQC NMR (800 MHz, $CDCl_3$) spectra of compound **10**.

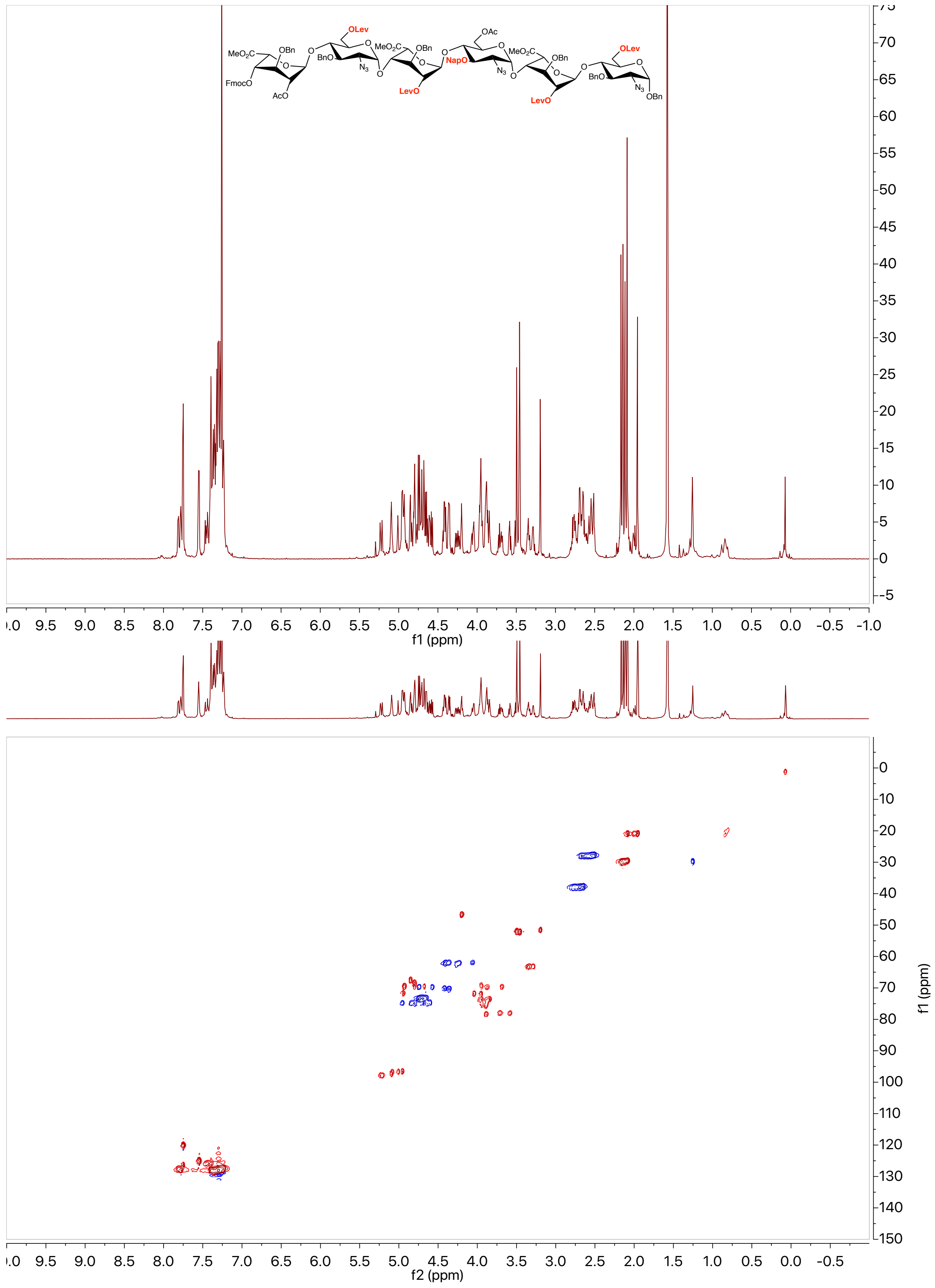

Figure S9. ^{1}H and HSQC NMR (500 MHz, $CDCl_3$) spectra of compound **11**.

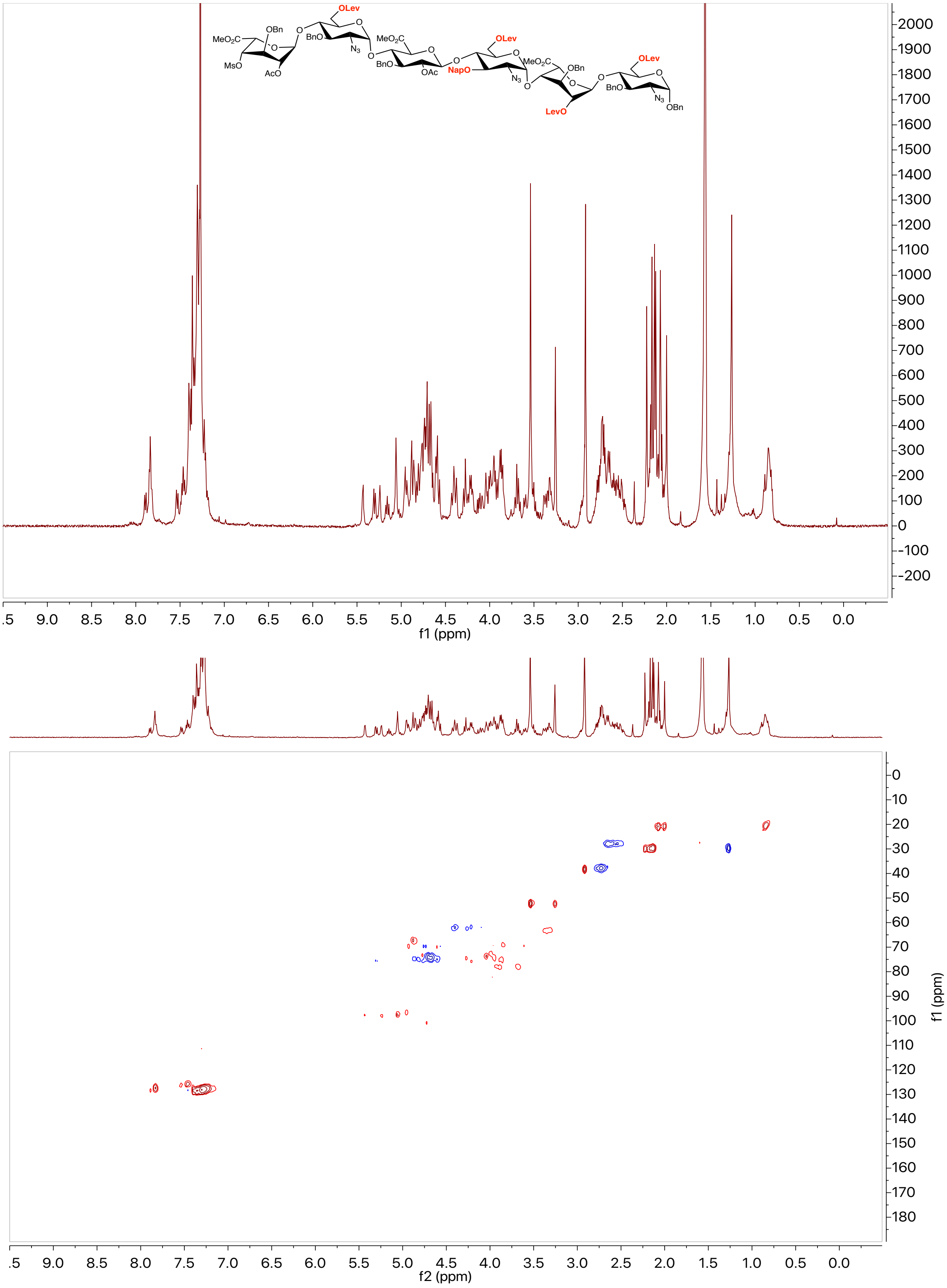

Figure S10. ^{1}H and HSQC NMR (600 MHz, $CDCl_3$) spectra of compound **12**.

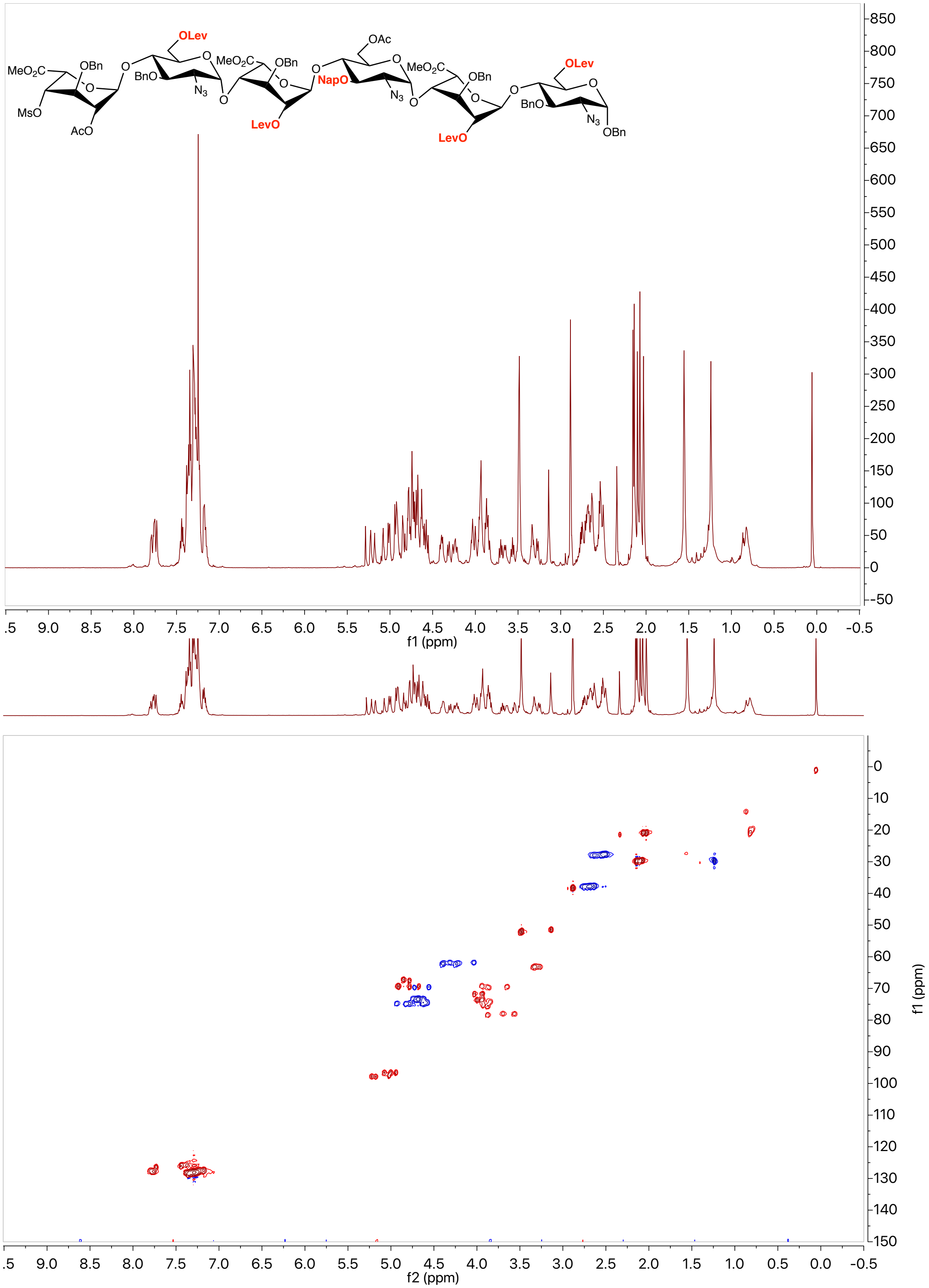

Figure S11. ^1H and HSQC NMR (600 MHz, D_2O) spectra of compound **13**.

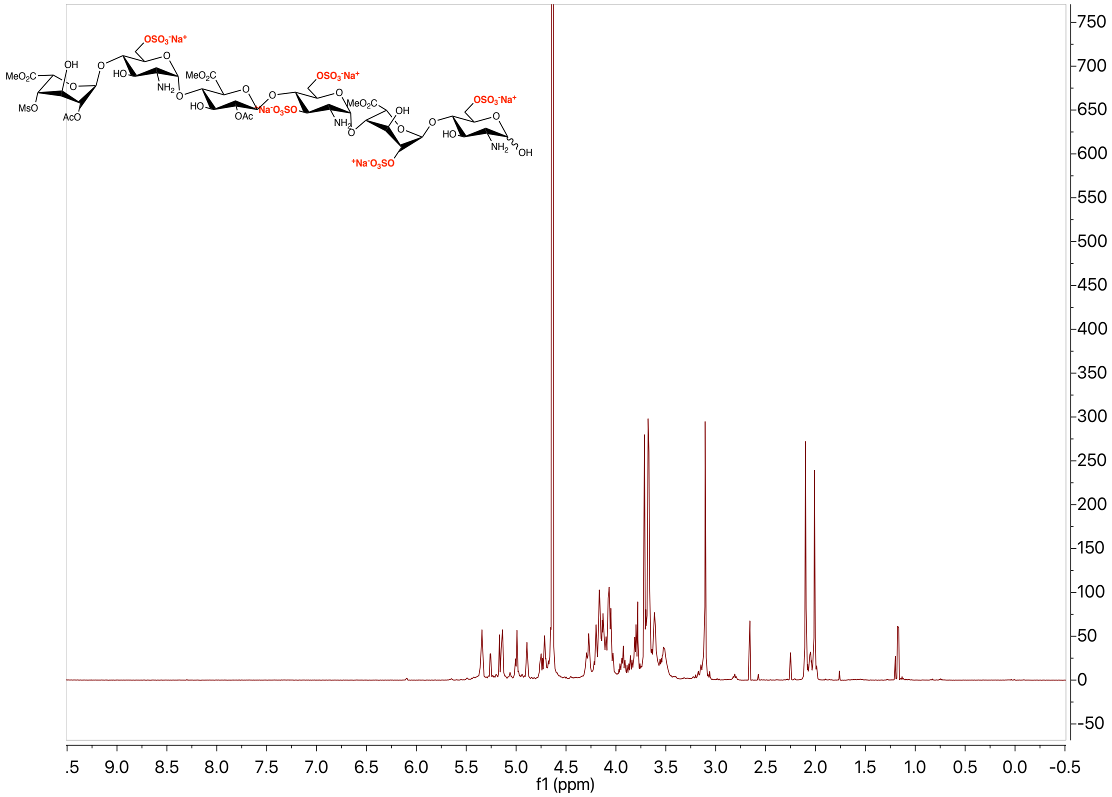

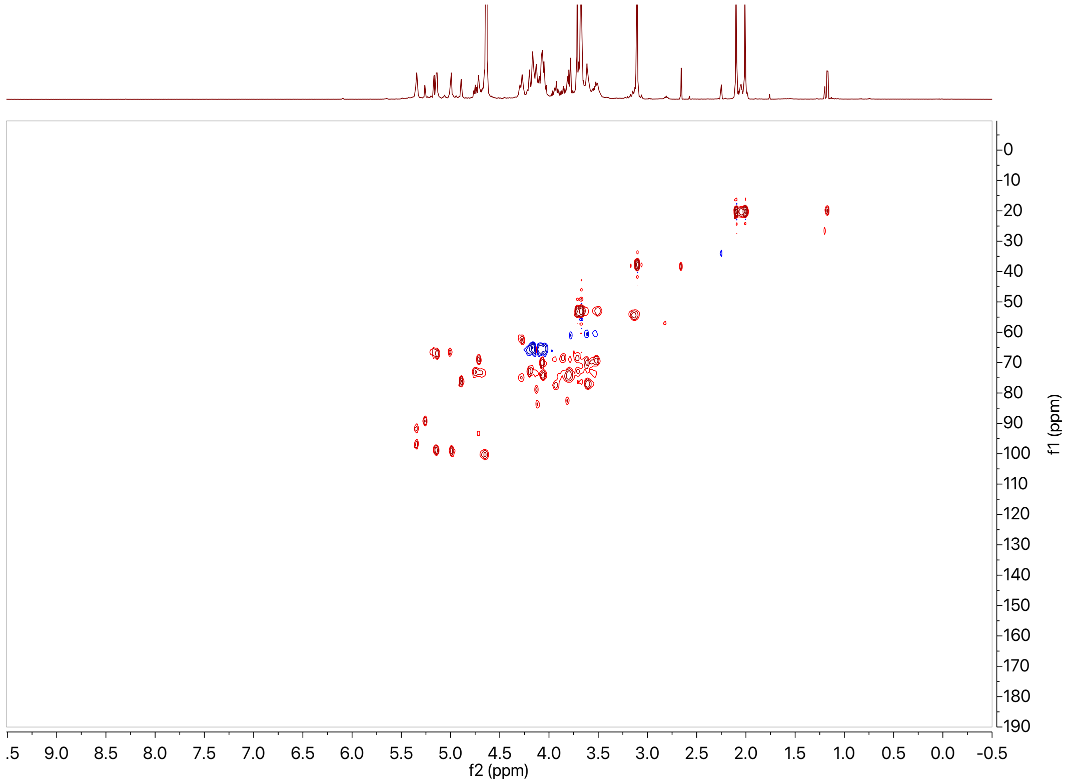

Figure S12. ^{1}H and HSQC NMR (500 MHz, D_2O) spectra of compound **14**.

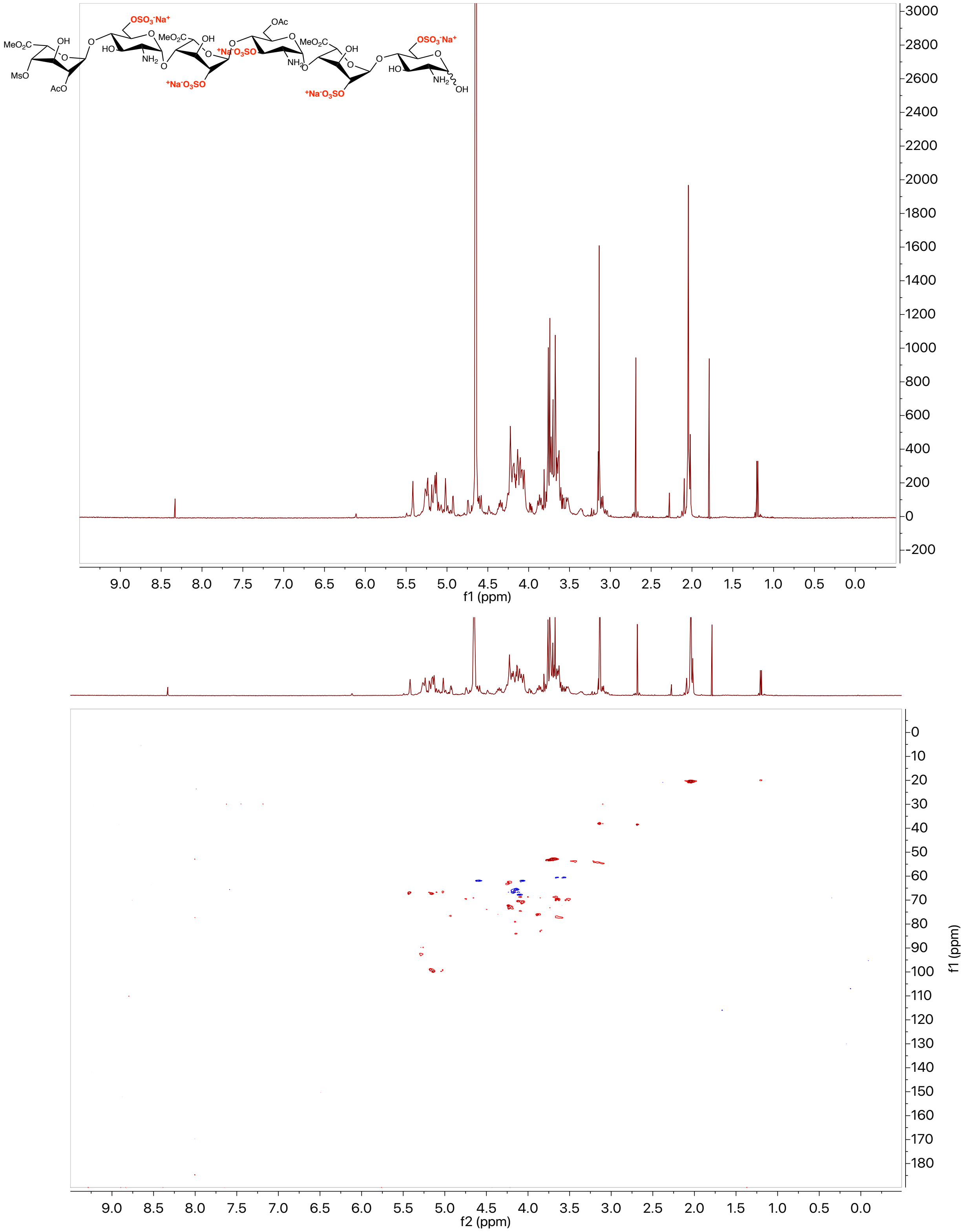

Figure S13. ^{1}H and HSQC NMR (600 MHz, D_2O) spectra of compound **1** (triethylamine salt).

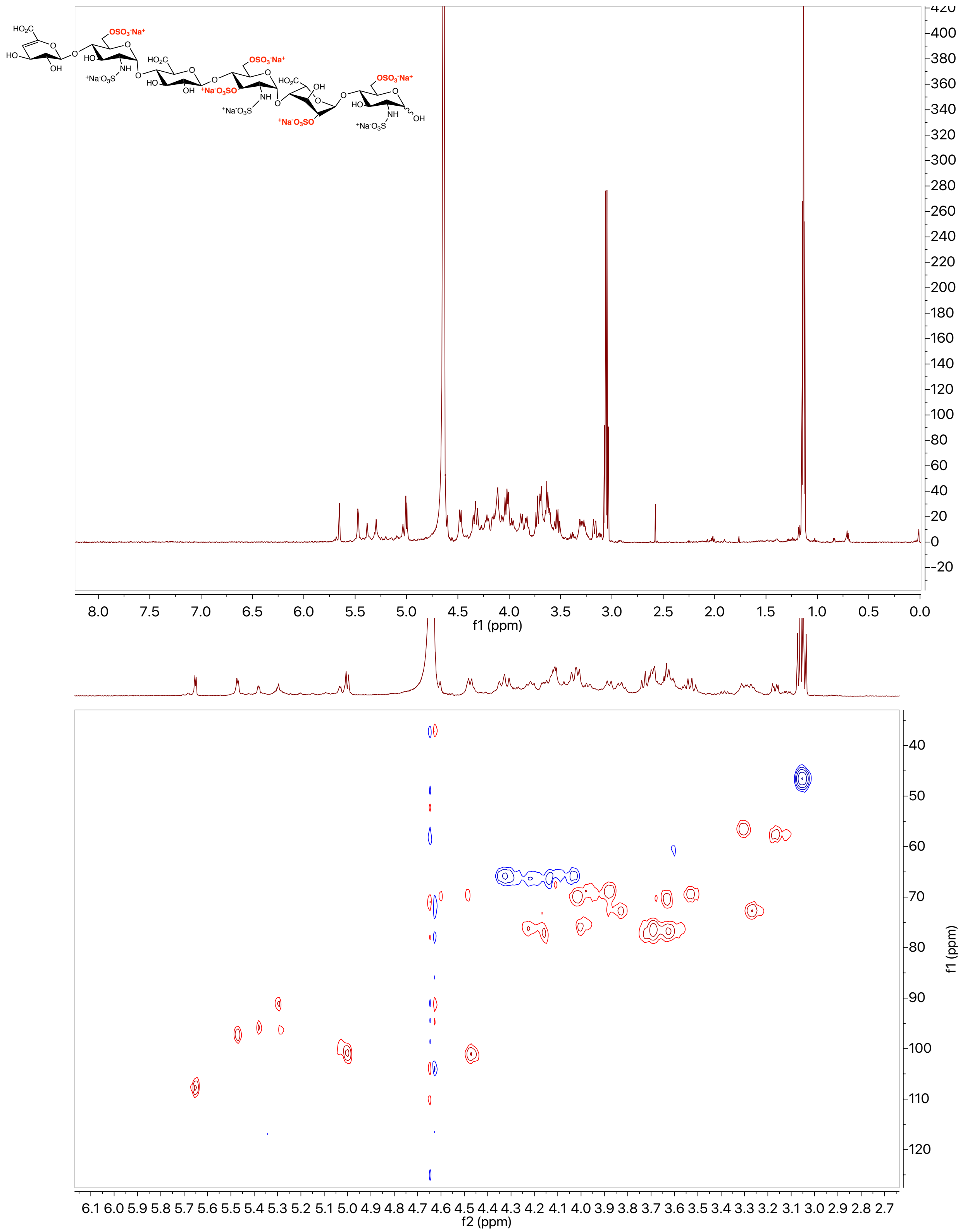

Figure S14. ^{1}H and HSQC NMR (600 MHz, D_2O) spectra of compound **2** (sodium salt).

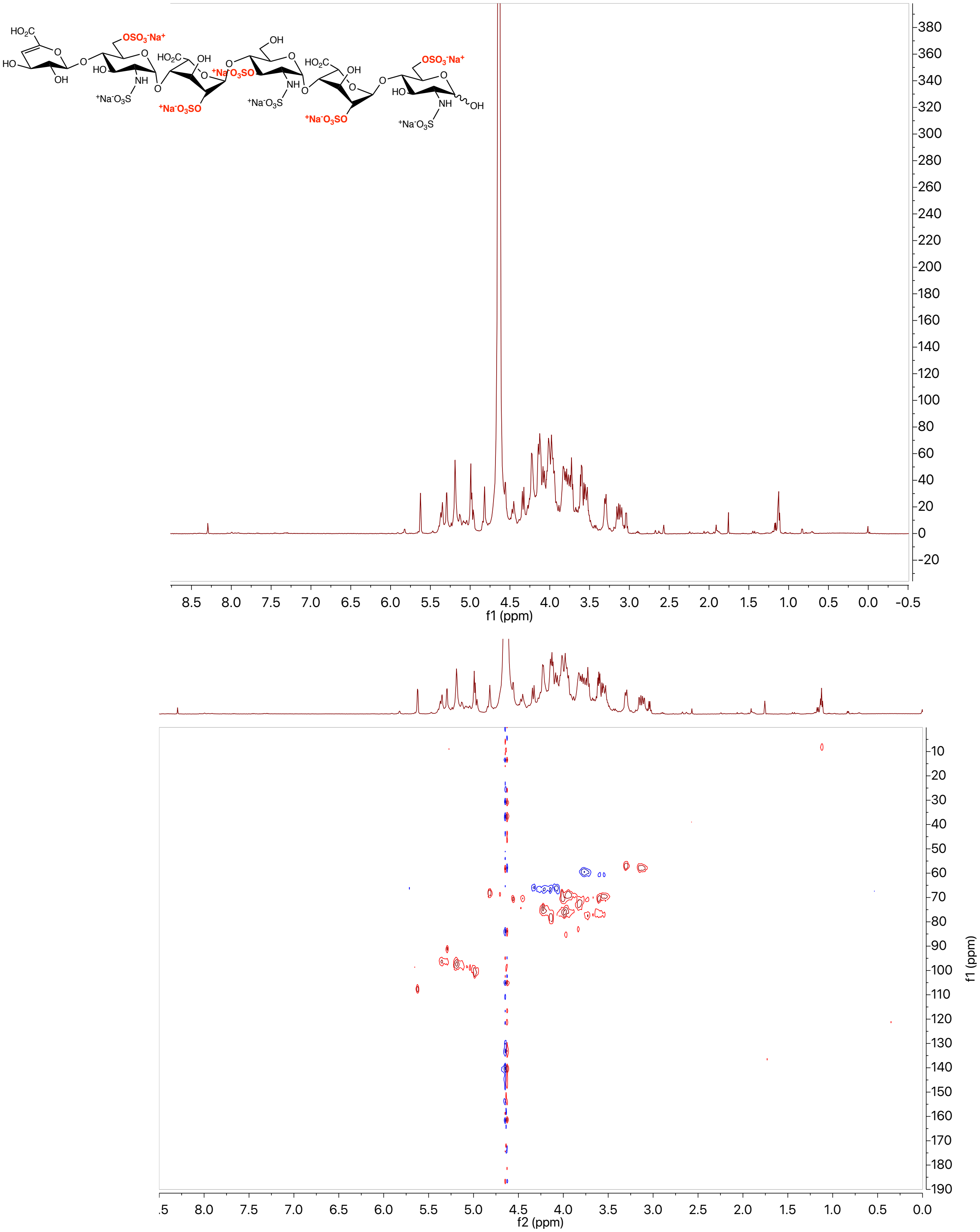

Supplement: Supplementary file 1 — ol4c00596_si_001.pdf [file ol4c00596_si_001.pdf]
